# Intermittent hypoxia ameliorates behavioral deficits and exerts neurorestoration in a mouse photothrombotic stroke model

**DOI:** 10.7150/thno.126126

**Published:** 2026-05-29

**Authors:** Tao Wu, Shu Feng, Mengjie Chen, Xin Wang, Emily Parker, Li Gong, Luodan Yang, Rui Duan

**Affiliations:** 1 School of Physical Education and Sports Science, South China Normal University, Guangzhou 510006, China.; 2 Jiangxi Science and Technology Normal University, Nanchang 330038, China.; 3 Medical College of Georgia, Augusta University, Augusta, GA 30912, USA.; 4 Experimental Animal Center, Nanfang Hospital, Southern Medical University, Guangzhou 510515, China.

**Keywords:** intermittent hypoxia, ischemic stroke, HIF-1α, glial polarization, mitochondria

## Abstract

**Background:**

Ischemic stroke disproportionately impacts the elderly with a higher risk and poor repair. Current therapeutic options are constrained by narrow time windows, strict contraindications, and suboptimal efficacy in older patients, leaving a critical unmet clinical need. Hypoxia-based interventions exert preclinical neuroprotective effects, yet the effects and underlying mechanisms of intermittent hypoxia (IH) in elderly patients with ischemic stroke remain unclear.

**Methods:**

This study tested IH in 25-month-old C57BL/6J mice with photothrombotic (PT) stroke, randomizing to Control, PT, or PT+IH groups. IH post-conditioning was administered a total of 7 sessions on days 3-16. One session consists of a 10-min phase with 8% oxygen, followed by a 10-min phase with room air. Behavioral changes were measured through the maximal grip strength test, beam balance test, cylinder test, adhesive removal test, grid-walking test, and open field test. Neuropathological changes and potential molecular mechanisms were analyzed via immunofluorescence staining and western blotting.

**Results:**

Our findings demonstrated that IH treatment significantly reduced cortical infarct volume and ameliorated PT-induced sensorimotor deficits in aged mice. Furthermore, IH alleviated neuronal damage and apoptosis, preserved cerebrovascular morphology, and attenuated excessive astrocyte-vasculature interactions. Mechanistically, IH upregulated astrocyte-specific hypoxia-inducible factor 1α (HIF-1α), mitigated mitochondrial fragmentation, and shifted the polarization of microglia and astrocytes from pro-inflammatory (M1/A1) to anti-inflammatory (M2/A2) phenotypes. Collectively, these effects contributed to enhanced neurogenesis and angiogenesis in the peri-infarct region.

**Conclusion:**

In conclusion, these findings confirm IH’s neurorestoration in aged stroke mice, potentially via HIF-1α-related regulation of mitochondrial function, glial polarization, and vascular integrity, supporting its translational potential.

## Introduction

Ischemic stroke remains a major contributor to enduring disability and mortality across the world, with its clinical impact exacerbated in aging populations, leaving survivors with motor coordination, locomotor deficits, and even cognitive disability 1. Compared to young adults, the elderly displayed a remarkably increased risk of ischemic stroke and compromised repair ability 1, 2. The dual challenges substantially increase the difficulty of treating stroke and facilitating rehabilitation in the elderly 1, 2. At present, the sole approved interventions for acute ischemic stroke are mechanical thrombectomy and intravenous tPA-based thrombolysis 3. Unfortunately, although both interventions focus on restoring blood flow to ischemic brain tissue, the narrow time windows, contraindications (e.g., hypertension, diabetes), and incomplete efficacy in elderly patients significantly limit the application for these patient subgroups 4, 5.

Ischemic insult, characterized by blocked or reduced cerebral blood flow (CBF), triggers progressive neuronal and tissue damage in the brain 6. The damaged brain tissues are mainly divided into two distinct regions, the peri-infarct area (also known as ischemic penumbra) and core infarct regions (or infarct core) 7. Surrounding the infarct core, the peri-infarct area is a zone where neurons retain structural integrity but exhibit functional impairment 8. Without timely therapeutic intervention, the peri-infarct area will progress to the infarct core, accompanied by irreversible neuronal death and glial cell damage. Improved functional recovery following treatment is closely associated with increased neurogenesis and angiogenesis within the peri-infarct zone 3, 9. Therefore, the peri-infarct area has been considered a promising targeted region for stroke therapies.

Hypoxia-inducible factor-1α (HIF-1α) is a sensitive intracellular oxygen sensor and transcription factor that regulates cellular adaptive responses to hypoxia 10. Accumulating evidence has indicated the potential neuroprotective effects of activation of HIF-1α following ischemic stroke 11. Under hypoxic conditions, the activation or improved expression of HIF-1α is essential in maintaining neurogenesis in the postnatal brain tissues 12. However, the approaches that improve HIF-1α accumulation or activation following ischemic injury displayed controversial outcomes. For example, increasing HIF-1α levels by deletion of the HIF suppressor or short hypoxic preconditioning has shown neuroprotective effects against ischemic brain injury 13, 14. However, other evidence has displayed opposite results. For instance, at the early stages of hypoxia, HIF-1α inhibition exhibited significantly elevated neuronal viability 15. Although controversial outcomes exist, approaches targeting HIF-1α still show great potential in the treatment of ischemic stroke.

Hypoxia, as the most direct approach that targets HIF-1α, has gained increasing focus in the treatment of brain disorders 16-18. Both hypoxic preconditioning and low oxygen post-conditioning with chronic continuous hypoxia (~7-11% oxygen) have shown promising results in improving functional recovery and exerting neuroprotection against ischemic stroke 17, 19. However, the therapeutic application of chronic continuous hypoxia, especially in elderly populations, which are the primary group affected by ischemic stroke, is limited by safety risks and the inadequacy of its beneficial effects 16, 20. These limitations underscore the need for safer hypoxic strategies. Studies employing the middle cerebral artery occlusion model have identified intermittent hypoxia (IH), which consists of alternating 7-11% oxygen and room air, as a candidate treatment for ischemic stroke 21. Existing research, utilizing a middle cerebral artery occlusion model, has provided initial evidence supporting its therapeutic potential 21. Despite this progress, critical gaps remain in our understanding of this approach. Specifically, it remains unclear whether the therapeutic effects of IH treatment can be replicated in other preclinical animal models of ischemic stroke. More importantly, there is a lack of evidence regarding its efficacy in elderly animal models, which are more clinically relevant given the high incidence of ischemic stroke in older adults. Additionally, the underlying neuroprotective mechanisms of intermittent hypoxia have not been fully elucidated, representing a key area for future investigation. Using a photothrombotic (PT) stroke animal model, we conducted experiments to investigate the effects of IH treatment on ischemic stroke-induced brain injury and functional deficits.

## Methods

### Animals and experimental design

**Figure 1:**
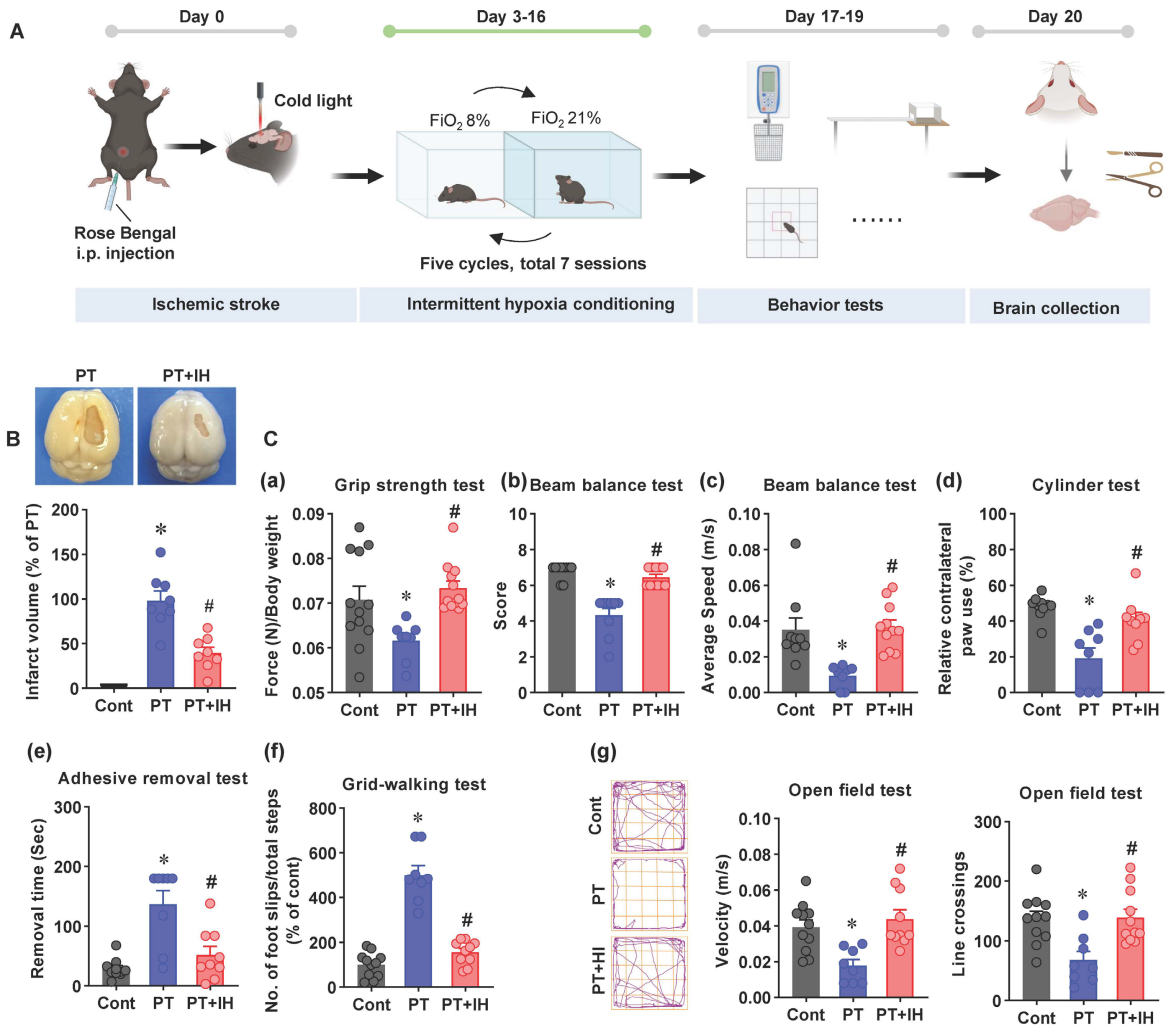
** Schematic depiction of the study timeline, infarct assessment, and behavioral testing in the PT stroke mouse model. (A)** Schematic illustration of the experimental timeline. On Day 0, mice were subjected to ischemic stroke via Rose Bengal and cold light induction. From day 3 to day 16, IH post-conditioning (seven sessions on days 3, 5, 7, 9, 11, 13, and 15; each session consists of five cycles of 10 min 8% FiO₂ / 10 min room air) was conducted, followed by behavioral test on days 17-19, and brain collections on day 20. **(B)** Representative images of brain infarct and quantification of infarct volume. *P < 0.05 *vs.* Cont; *^#^P* < 0.05 *vs.* PT. **(C)** Behavioral assessments: (a) Grip strength test, with force normalized to body weight (N/body weight); (b) Beam balance test (score); (c) Beam balance test (average speed, m/s); (d) Cylinder test (relative contralateral paw use, %); (e) Adhesive removal test; (f) Grid-walking test; (g) Open field test: Representative escaping traces of the animals and analysis on velocity and line crossings. FiO_2_: Fraction of inspired oxygen. Data shown as mean ± SEM. n = 8-12. *P < 0.05 *vs.* Cont; *^#^P* < 0.05 *vs.* PT.

Male C57BL/6J mice, initially 3 months old, were obtained from the Guangdong Medical Laboratory Animal Center (Guangzhou, China). They were then housed in a central facility under a 12-h light/dark cycle (22 ± 2 °C, 50 ± 10% humidity) and aged naturally to 25 months. Standard chow and filtered water were provided *ad libitum*, with no other dietary or water supplements. At 25 months, mice were randomly assigned to 3 groups (n = 8-12 per group): (1) Control (no treatment); (2) PT stroke; (3) PT+IH. IH intervention was initiated on post-stroke day 3 to target the subacute reparative phase, thereby avoiding potential interference with acute pathological events within the first 72 hours 22, 23. As shown in **Figure 1A**, IH treatment was performed following previous studies with minor modifications 24, 25. IH post-conditioning was administered for a total of seven sessions, conducted every other day from day 3 to day 15 post-stroke (specifically on days 3, 5, 7, 9, 11, 13, and 15). Each session consisted of five cycles, with each cycle comprising 10 min of 8% FiO₂ followed by 10 min of room air (21% FiO₂). For IH exposure, mice were placed in a custom-built Plexiglas chamber (volume: 5.4 L) with a constant gas flow rate of 3.5 L/min, controlled by an O₂ programmable gas controller (ProOx 110, BioSpherix, NY, USA). The time required to switch from room air (21% O₂) to 8% O₂ within the chamber was approximately 45 s (lag time). Animals were exposed to IH in groups of 3-4 per chamber, with bedding provided to minimize stress. Food and water were not available during the 100-min IH sessions. The chamber was cleaned and dried between sessions to prevent ammonia buildup. Behavioral tests were conducted on days 17-19 following IH treatment. Mice were then euthanized for brain collection. All procedures were approved by the IACUC of South China Normal University (SCNU-SPT-2025-016) and complied with NIH’s Guide for the Care and Use of Laboratory Animals.

### PT stroke model

The PT stroke animal model was established by integrating our previously described protocol with well-established standard methods 9, 26, 27. In brief, we anesthetized the mice with an intraperitoneal injection of sodium pentobarbital (50 mg/kg body weight). Following confirmation of deep anesthesia, animals were fixed in a stereotactic frame to maintain skull position. Subsequently, Rose Bengal dye (0.1 mg/g body weight, within the effective range of 50-150 µg/g reported for photothrombotic protocols 27) was administered *i.p.*, and a 5-min interval was allowed for dye circulation. The skull was exposed via a scalp incision, and the periosteum was gently stripped away to permit clear light transmission. A 6-mm cold white light beam connected to a fiber optic cable was placed on the skull at 1.8 mm anterior to bregma and 2.5 mm lateral to the midline, with the light source’s distance and angle fixed across animals for reproducible lesion induction. The illumination intensity was calibrated to 0.1 W/cm² at the skull surface, as validated in classic photothrombotic stroke studies 28, 29. The target skull region was illuminated continuously for 15 min to induce local photothrombosis. This protocol has been successfully applied in aged (24-month-old) C57BL/6 mice 26, confirming its suitability for the aged cohort used in the present study.

### Behavioral tests

#### Maximal grip strength test

To assess forelimb grip strength, we employed a grip strength test according to our previously published protocol 30. A digital grip-strength meter was used to measure forelimb grip strength. For each experimental subject, the grip strength assessment was conducted in five trials to account for potential variability in performance. Among the five recorded measurements, the highest value was recorded as the maximal grip strength. To eliminate the confounding effect of body mass on strength readings, the selected maximal grip strength was subsequently normalized to the subject’s body weight and expressed as grip strength per unit body weight.

#### Beam balance test

To assess motor coordination and balance, we performed the beam balance test as previously described 31, 32. A balance beam measuring 100 cm long and 7 cm wide was placed at a height of 100 cm above the floor. Mice were placed at the beam’s far end and permitted to walk across and back to their home cage. Completion time was recorded, and average speed was computed. In addition, a 7-point scale quantifies performance according to a previous study 33, which adapted this scoring system from the rat literature for use in mice 32, from 7 (smooth crossing, no deficits) to 1 (can’t crawl, falls off), with 3 trials averaged/median-scored for analysis. The detailed 7-point scale is as follows: 7, The mouse crosses the balance beam smoothly; the potentially impaired limb functions completely normally, with no obvious signs of neurological damage; 6 points, the mouse crosses the balance beam, and the functional contribution of the potentially impaired limb is >50%; 5, The mouse crosses the balance beam, and the functional contribution of the potentially impaired limb is < 50%; 4 points, The animal is unable to traverse the beam without difficulty, and its fall frequency (falls divided by total attempted steps) remains below 50%; 3 points, the mouse fails to cross the balance beam smoothly; the fall rate is > 50%; 2, the mouse is unable to move forward on the balance beam but can maintain a sitting posture on the beam surface; 1, the mouse is completely unable to crawl on the beam; additionally, it cannot maintain the hindlimbs in a horizontal position on the beam surface, and will fall off the beam if placed on it.

#### Cylinder test

To evaluate how IH treatment influences asymmetric forelimb use, we performed the cylinder test as described in our earlier work 9. A clear glass cylinder measuring 10 cm in diameter and 15 cm in height served as the test apparatus. Each animal was placed individually inside the cylinder, and a 2-minute observation period was conducted to record forelimb-wall interactions. The number of the left and right forelimbs (paws) in contact with the inner wall of the glass cylinder was manually counted by a researcher blinded to the treatment groups. The following calculation was applied to quantify the degree of contralateral forelimb use, a key indicator of motor asymmetry post-stroke: Relative contralateral paw use = (number of contralateral paw contacts / total number of paw contacts) × 100.

#### Adhesive removal test

Somatosensory deficits were evaluated using the adhesive removal test, a common behavioral assay 9. Briefly, prior to each trial, two adhesive strips (0.35 × 0.45 cm) were placed on the inner side of each front paw to provide bilateral somatosensory stimulation. Following strip application, the mouse was housed singly in its home cage, and the removal time for each forepaw strip was recorded, not exceeding 3 minutes.

#### Grid-walking test

The grid-walking test is a common assay to evaluate rodent motor function and limb coordination 34. Animals were individually placed on an elevated wire grid with species-specific square openings (1.69 cm² for mice) and allowed to traverse it. The number of foot slips (a paw fell through or slipped off the grid’s wire frame) and the total steps to cross the grid were recorded and analyzed.

#### Open field test

The open field test, a widely used rodent behavioral assay for locomotion and exploration, was performed as per our prior protocol 35. A black square box (40 × 60 × 60 cm) served as the open field. Mice were placed singly at the center for 5 min of free exploration. The apparatus was cleaned with 70% ethanol between trials to avoid cross-contamination. ANY-maze software automatically tracked locomotor and exploratory parameters; line crossings and velocity were analyzed.

All behavioral experiments and their analyses were performed by an experimenter masked to the group allocation. For each test, the scoring of video recordings (e.g., beam balance score, foot slips, adhesive removal time) was performed offline by a second blinded investigator to ensure objectivity. For each behavioral test, three consecutive trials were conducted per animal, and the average score was used for statistical analysis (for the beam balance test, the median score of three trials was used). Animals that fell more than twice during a test session or failed to initiate movement within two minutes were excluded from that specific test analysis. All data are presented as mean±SEM in the figures.

### Brain collection and tissue preparation

After the behavioral tests on day 20 after the PT stroke, we harvested the brains and prepared the tissues. Following the protocol detailed in our earlier work 36, Brains were quickly removed following transcardial perfusion with ice-cold saline. For histology, harvested brains were fixed overnight in 4% PFA at 4 °C, then cryoprotected in 30% sucrose until the tissue sank. The brains were then embedded in OCT and stored at -80 °C overnight. Coronal sections (25 μm) were cut on a Leica RM2155 cryostat and kept in antifreeze solution (PC101, FD NeuroTechnologies) for long-term storage. For protein studies, the peri-infarct cortex and corresponding regions were microdissected immediately after brain removal and snap-frozen in liquid nitrogen. Homogenization followed our previously described protocol 37. Brain samples were homogenized with a motor-driven Teflon homogenizer in ice-cold buffer containing 50 mM HEPES (pH 7.4), 150 mM NaCl, 12 mM β-glycerophosphate, 1% Triton X-100, plus protease and phosphatase inhibitors (Thermo Scientific, Rockford, IL). After homogenization, the samples were incubated under gentle agitation for 20 min, then centrifuged at 12,000 × g for 30 min at 4 °C to collect total protein fractions.

### Immunofluorescence staining

Immunofluorescence staining was carried out according to procedures established in our prior publication 38. Brain slices obtained by the protocol above were permeabilized for 4 h in 0.4% Triton X-100, then blocked for 60 min with a buffer containing 10% normal donkey serum. The slices were subsequently incubated with primary antibodies (listed in **Table S1**) overnight at 4 °C. After three washes, the sections were incubated with the appropriate Alexa Fluor-conjugated donkey secondary antibodies (anti-mouse/rabbit/goat) for 30 min at room temperature. Following a final wash series, the sections were mounted with DAPI Fluoromount-G® (SouthernBiotech) to counterstain nuclei and retain fluorescence. Imaging was performed using a Zeiss LSM800 confocal laser scanning microscope. All image acquisition was carried out by an investigator masked to experimental group assignments, with identical laser power, gain, and exposure settings for each target across all samples.

### Quantification of cortical infarct volume and synaptic markers

For cortical infarct volume quantification (performed on a separate cohort of 8 mice per group), MAP2 immunofluorescence was used to delineate the infarct core. Whole coronal sections (30 µm thickness, collected every 180 µm) were imaged under the confocal microscope. The infarct area was defined as a sharply demarcated region showing complete loss of MAP2 signal (MAP2-negative). For each section, the MAP2-negative area was manually traced by an investigator blinded to group allocation using FIJI-ImageJ. Total infarct volume (mm³) was calculated by summing the MAP2-negative areas from all sampled sections and multiplying by the sampling interval (180 µm). The infarct volume of each sample was then expressed as a percentage of the mean infarct volume of the PT group.

Using ImageJ software (NIH, USA) and the “Puncta Analyzer” plugin, we counted spinophilin-positive, synaptophysin-positive, and colocalized puncta in at least three randomly selected 50 × 50 µm regions per section from the peri-infarct area. Confocal images were acquired with a Zeiss LSM800 microscope (63 × oil-immersion objective), following the protocol of a previous study 39. Results from 8 animals per group were averaged and normalized to the control group as percentage changes. Spinophilin is a well-established marker for dendritic spines (postsynaptic density), and synaptophysin is a marker for presynaptic vesicles. Colocalized puncta represent apposed pre- and postsynaptic structures, thus reflecting functional synaptic density and potential transmission efficiency 39.

### 5-bromodeoxyuridine (BrdU) labeling

BrdU (5-bromo-2’-deoxyuridine, Sigma-Aldrich) was prepared in sterile saline and given via intraperitoneal injection at a dose of 50 mg/kg, as described in an earlier study 40. To label proliferating cells induced by IH treatment, BrdU was injected twice daily (every 12 h) for three consecutive days (days 16-18 post-stroke), immediately following the last IH session. Mice were euthanized 48 h after the final BrdU injection (day 20 post-stroke), allowing sufficient time for BrdU incorporation and subsequent differentiation of newborn neurons. This short-term, high-frequency labeling regimen is widely used to detect therapy-induced neurogenesis in stroke models, as it minimizes interference from the intervention itself and provides a clear temporal window for assessing newly generated neurons.

### Quantification of ATP levels

ATP levels were measured using an ENLITEN luciferin/luciferase kit (Promega, FF2021) following previous studies 41, 42. Briefly, ATP levels were measured by mixing 30 μg total protein with 100 μL reaction buffer (luciferase, D-luciferin, Tris-acetate pH 7.75, EDTA, magnesium acetate, BSA, DTT). Luminescence was read every 10 s in a microplate luminometer (PE Applied Biosystems). A blank (buffer without protein) was subtracted from each sample, and ATP concentrations were interpolated from a standard curve.

### Evans blue extravasation assay

Evans blue dye (50 mg/kg, Sigma-Aldrich, E2129) was injected intravenously (tail vein) to assess BBB permeability. After 4 h of circulation, mice were perfused with ice-cold saline to clear intravascular dye. Brains were rapidly removed, and the peri-infarct cortex was dissected. Tissue samples (30 μg protein) were homogenized, and Evans blue was quantified using a Perkin-Elmer fluorescence spectrophotometer (620 nm excitation/680 nm emission) according to a previous study 3.

### Image processing and analysis

Image analysis was performed with ImageJ (v1.49, NIH) or Imaris (Bitplane AG). Quantification of target signal intensity and microglial morphology via Sholl analysis was conducted using Fiji/ImageJ (v2.9.0, NIH) as reported earlier 35, 43. Three-dimensional reconstruction of high-resolution Z-stack images was carried out with Imaris software (v9.7.0, Bitplane AG), following the protocol from our previous work 37. The “Surface” detection module was selected, and a consistent surface detail parameter of 0.4 μm was applied across all images. Nonspecific signals and background noise were eliminated by setting appropriate intensity thresholds. Mitochondrial analysis was performed with Fiji ImageJ (v2.9.0, NIH) to count total, small (< 1.5 μm), and continuous (> 2 μm) particles, as previously reported 42. Particle counts (total, small, and continuous) were expressed relative to the total mitochondrial area. For microglial morphology, we performed Sholl analysis with Fiji-ImageJ (v2.9.0, NIH, Bethesda, MD, USA) following a protocol from our prior work. Branch length, microglial cell diameter, number of branches, and intersections with concentric circles were used to characterize microglial morphological changes 35.

### Western blotting analysis

Western blot analysis was carried out according to procedures established in our prior publication 44. Total proteins were denatured in SDS sample buffer at 95 °C for 10 min. A uniform amount (30-50 μg per lane) was separated by 10-12% SDS-PAGE under constant voltage (120 V, 90 min). The resolved proteins were transferred to PVDF membranes via a semi-dry transfer system. Membranes were then blocked with 3% BSA for 30 min at ambient temperature, followed by overnight incubation at 4 °C with the primary antibodies in blocking buffer. After three 10-min TBST washes, the membranes were incubated with HRP-linked secondary antibodies for 1 h at room temperature. Protein bands were detected using ECL reagent and imaged with a cold CCD system. Band intensities were analyzed with ImageJ (v2.9.0, NIH), with β-actin or α-Tubulin used as the internal control for normalization.

### Reverse transcription quantitative PCR

RNA was extracted from peri-infarct tissue using RNAiso Plus (Takara #9109). Tissue was homogenized in 1 mL of the reagent, incubated 5 min at RT, and centrifuged (12,000×g, 5 min, 4 °C). The supernatant was mixed with 200 μL chloroform, vortexed, incubated for 5 min, and centrifuged (12,000×g, 15 min, 4 °C). The aqueous phase was precipitated with 600 μL isopropanol (10 min, then 12,000×g, 10 min, 4 °C). The pellet was washed twice with 75% ethanol (7,500×g, 5 min, 4 °C), air-dried, and dissolved in DEPC water. RNA was diluted to 100 ng/μL. Reverse transcription (PrimeScript™ RT Kit, random primers + Oligo dT) was run at 37 °C for 15 min, 85 °C for 5 sec. qPCR (TB Green Premix Ex Taq™, Bio-Rad) used triplicate reactions with cycling: 95 °C 30 s; 40× (95 °C 5 s, 60 °C 30 s); melt curve. Target genes were normalized to an internal reference and expressed relative to the control (set to 1). Primers are listed in **Table S2** (designed with NCBI Primer-BLAST).

### Statistical analysis

Data analysis was conducted with GraphPad Prism (v8.3.0). Comparisons across the three groups (Cont, PT, IH) were evaluated by one-way ANOVA. Pairwise comparisons of interest (Cont *vs.* PT and PT *vs.* IH) were conducted using the Holm-Šídák method to correct for multiple comparisons. Effect sizes (η² for ANOVA, Cohen’s d for pairwise comparisons) and 95% confidence intervals for the relevant comparisons are reported in **Table S3**. All data are presented as mean ± standard error of the mean (SEM). In all analyses, “n” refers to the number of mice per group (biological replicates). For histological analyses, multiple sections per mouse were averaged, and “n” indicates the number of mice. Sample sizes for the aged cohorts were determined a priori using G*Power (effect size = 0.80, α = 0.05, power = 0.80), yielding a minimum of n = 8 mice per group (see **Table S4** for detailed input parameters and output). Sample sizes varied by assay: 8-12 mice/group for behavioral tests; 8 mice/group for histology and most molecular analyses (brain sections/ROIs); 4 or 8 mice/group for Western blot; and 3 mice/group for RT-qPCR. A p-value <0.05 was considered significant.

## Results

### IH treatment ameliorates PT stroke-induced cortical damage and behavioral deficits

To investigate the effects of IH treatment on cortical damage, we first analyzed the cortical infarct volume following brain collection. As shown in **Figure 1B**, considerable cortical damage was present in the right somatosensory cortex of PT animals, whereas animals treated with IH displayed significantly reduced cortical infarct volume. We also measured the effects of IH treatment on PT stroke-induced behavioral changes using a battery of behavioral tests. As shown in **Figure 1C**(**a**), the grip strength was significantly decreased compared with that of the control animals. In contrast, the impaired grip strength was significantly ameliorated. In addition, the beam balance tests (**Figure 1C**(**b&c**)), the cylinder test (**Figure 1C**(**d**)), the adhesive test (**Figure 1C**(**e**)), and the grid-walking test (**Figure 1C**(**f**)) were performed to test the ability of muscle control and body balance, sensorimotor dysfunction, the sensorimotor integration ability of the affected limb, and motor skills and spatial orientation ability of animals, respectively. Data analysis demonstrated that PT-induced stroke led to marked behavioral impairments across all these functional domains. Promisingly, IH treatment exerted a robust corrective effect on these PT-induced behavioral impairments. Finally, the open field test was performed to evaluate the locomotor activity. As shown in **Figure 1C**(**g**)**,** animals subjected to PT stroke present a significant decrease in velocity and line crossings as compared with the controls. In contrast, IH treatment promotes the recovery of locomotor ability, as evidenced by significantly increased velocity and line crossing. Collectively, these behavioral data, combined with the earlier findings on cortical infarct volume reduction, strongly support that IH treatment significantly mitigates cortical damage and sensorimotor and motor coordination deficits following ischemic stroke.

### IH treatment protects against PT stroke-induced neuronal apoptosis and damage

**Figure 2:**
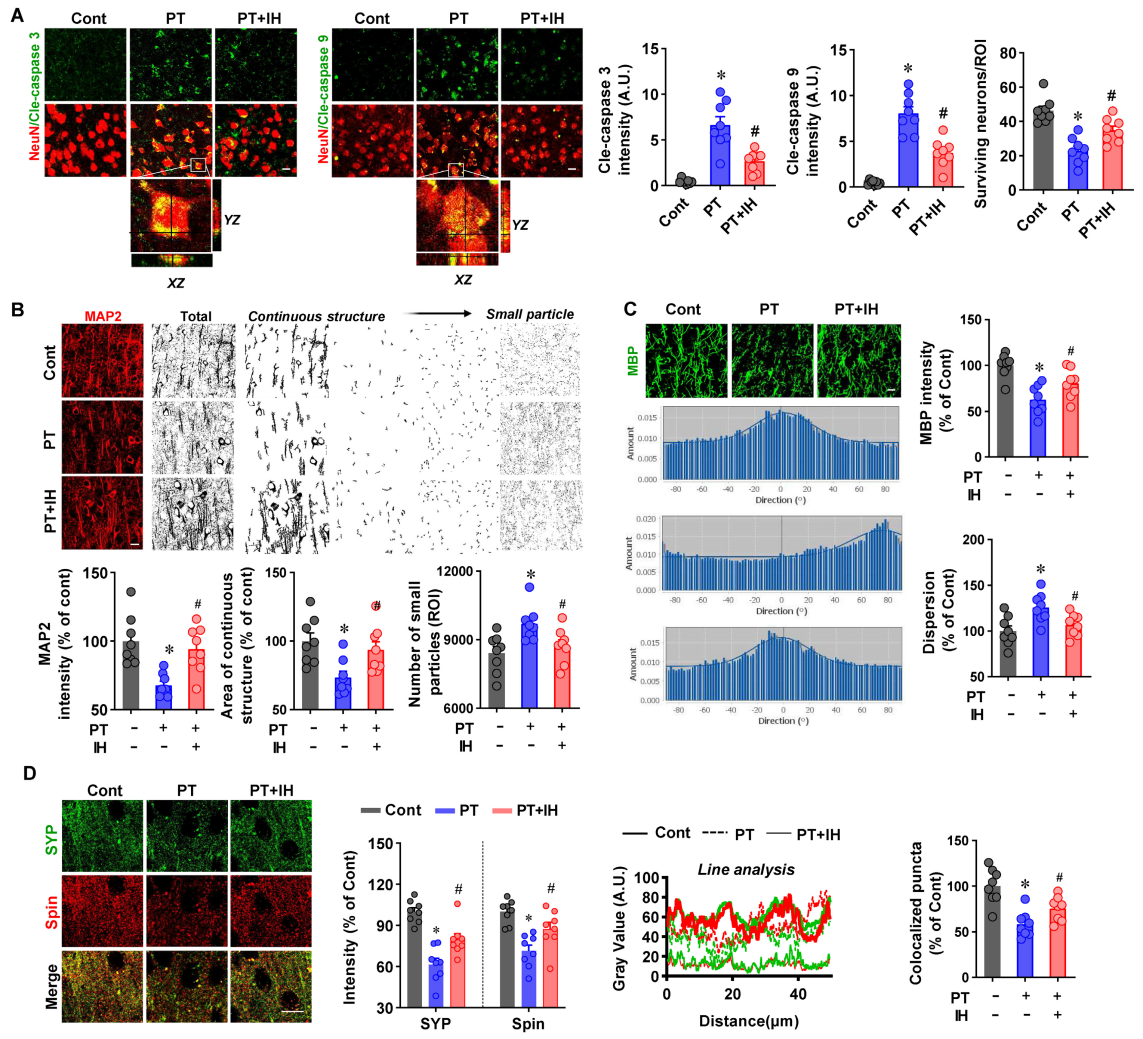
** IH alleviates PT stroke-induced neuronal apoptosis and injury. (A)** Representative images of NeuN co-stained with cleaved caspase-3 and cleaved caspase-9 are presented. A confocal orthogonal magnification of the white inset (typical PT group) is shown. We quantified cleaved caspase-3/9 intensity and the number of surviving neurons. **(B)** Representative immunofluorescence images of MAP2. MAP2 signals were categorized as either continuous networks or fragmented particles of different sizes, and the number of particles in each category was quantified. **(C)** Representative immunofluorescence images of MBP, directionality histograms of MBP, and quantification of MBP intensity and dispersion are presented.** (D)** Immunofluorescence staining of synaptophysin (SYP, a presynaptic marker), spinophilin (Spin, a postsynaptic marker), and merged images are shown. Quantification of SYP and Spin intensity, colocalized puncta, along with line analysis of gray value, was carried out. Scale bar=10 μm. Data are presented as mean ± SEM. n = 8. *P < 0.05 *vs.* Cont; *^#^P* < 0.05 *vs.* PT.

To further investigate the protective effects of IH treatment against PT stroke-induced neuronal injury, we first evaluated neuronal apoptosis and survival by performing immunofluorescence staining of brain slices with NeuN (a neuronal marker) and cleaved-caspase-3/9 (apoptosis-related markers), followed by quantification of fluorescent intensity and the number of surviving neurons. As illustrated in **Figure 2**, PT stroke significantly upregulated cleaved-caspase-3 and cleaved-caspase-9 fluorescence compared with control and IH groups. Line scan analysis verified prominent colocalization of NeuN and cleaved caspase-3 within the peri-infarct region following PT treatment. PT insult also markedly reduced surviving neuron counts, an effect reversed by IH treatment.

The effect of IH on PT stroke-induced neuronal injury was further explored using neuronal damage markers: MAP2 (dendrites), MBP (myelin sheaths), SYP (presynaptic), and spinophilin (postsynaptic). PT stroke caused both a reduction in MAP2 fluorescence intensity and extensive MAP2 fragmentation in the peri-infarct area (**Figure 2B**). The fragmentation was reflected by a decrease in continuous MAP2 structures and an increase in small MAP2-positive particles. IH treatment effectively reversed the loss of MAP2 intensity and attenuated the fragmentation.

Moreover, as shown in **Figure 2C**, PT stroke insults significantly reduced MBP fluorescent intensity and notably increased MBP dispersion. Interestingly, IH treatment preserved MBP fluorescent intensity and significantly decreased MBP dispersion compared with the PT stroke group. Similarly, as illustrated in **Figure 2D**, compared with the control group, PT stroke significantly decreased the fluorescent intensity of Spin and SYP, as well as the number of Spin-SYP colocalized puncta (a marker of functional synapses). In contrast, IH treatment effectively ameliorated these PT stroke-induced reductions.

### IH treatment preserves vascular morphology and alleviates excessive astrocyte-vasculature interactions

**Figure 3:**
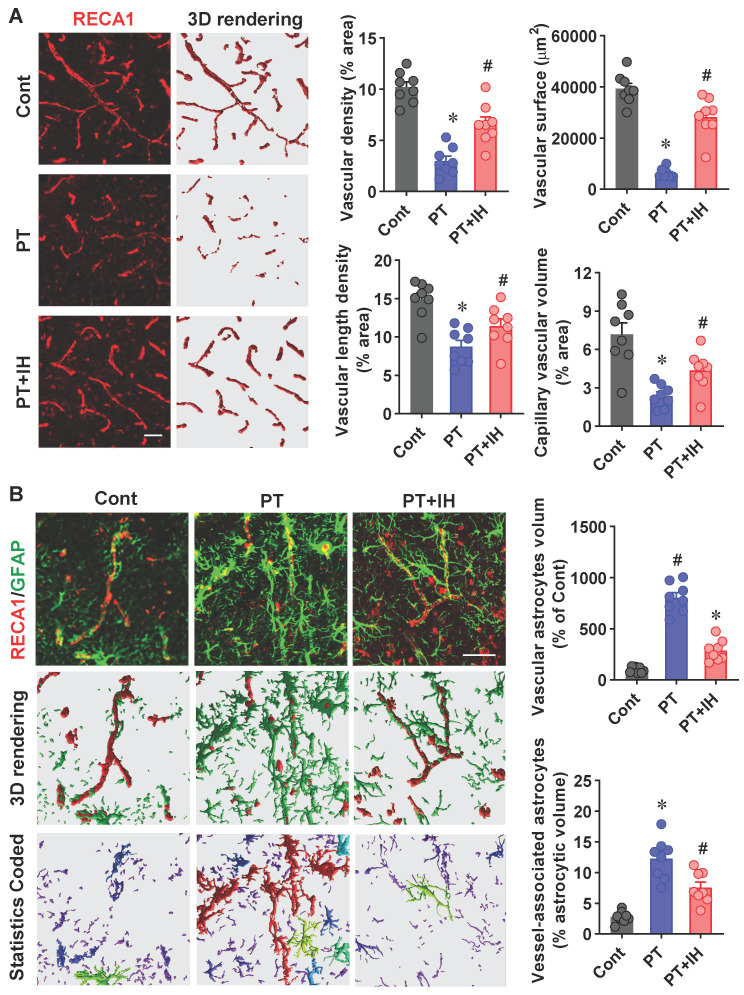
** IH treatment preserved vascular morphology and alleviated excessive astrocyte-vasculature interactions. (A)** Representative RECA1 immunofluorescence images and 3D reconstructions of vascular networks from the Cont, PT, and PT + IH groups. Quantitation of vessel density, length density, surface area, and capillary volume. **(B)** Representative RECA1 (red) and GFAP (green) images, 3D rendering of vascular and astrocyte structures, and statistics-coded images (different colors represent individual astrocytes). Quantification of vascular astrocyte volume and vessel-associated astrocytic volume. Scale bar=50 μm. Data are presented as mean ± SEM. n = 8. *P < 0.05 *vs.* Cont; *^#^P* < 0.05 *vs.* PT.

The cerebrovascular morphological alterations have been detected in ischemic stroke, and vascular remodeling after ischemic stroke is involved in ischemic stroke recurrence and neural function 45. To evaluate the role of IH treatment in post-stroke cerebrovascular homeostasis, we first analyzed the structural characteristics of the cerebral vasculature and the interaction between astrocytes and blood vessels in the peri-infarct regions. As shown in **Figure 3A,** vascular morphology was compromised following PT stroke compared to control animals, as evidenced by decreased vascular density, surface area, vascular length density, and capillary vascular volume. IH treatment alleviates vascular morphological alterations. Critically, to confirm that these structural improvements translate to functional vascular protection, we further assessed BBB permeability via Evans Blue extravasation assay. As demonstrated in **Figure S1**, PT stroke induced a marked increase in Evans Blue leakage in the peri-infarct region relative to control mice, indicating severe BBB disruption. Notably, IH treatment significantly reduced Evans Blue extravasation compared to PT mice, directly verifying that the observed vascular morphological improvements are associated with preserved BBB integrity.

In addition, excessive astrocyte-vasculature interactions have been detected following PT stroke, which hampered vascular repair according to a previous study 3. Similarly, as shown in **Figure 3B**, 3D reconstruction of the astrocyte (GFAP, green) and vascular (RECA1, red) showed significantly increased astrocyte volume and excessively elevated astrocyte-vasculature interactions. By contrast, IH treatment attenuates PT-stroke-induced excessive activation of glial cells and astrocyte-vasculature interactions.

### IH treatment promotes astrocyte-specific HIF-1α expression and preserves mitochondrial integrity and function

**Figure 4:**
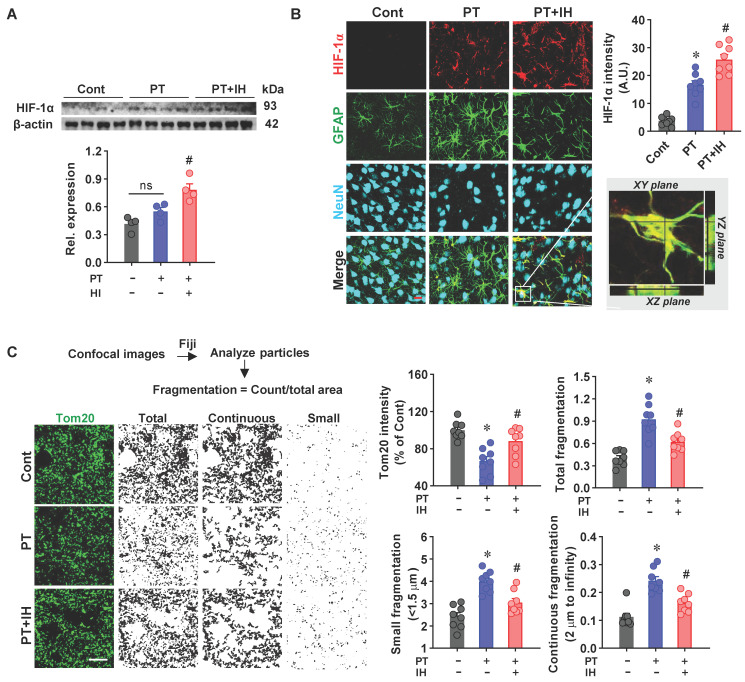
** IH treatment promoted astrocyte-specific HIF-1α expression and ameliorated mitochondrial fragmentation.** (A) Western blot analysis of HIF-1α expression with β-actin as a loading control. Quantitative analysis of relative HIF-1α expression. n = 4. **(B)** Representative immunofluorescence images of HIF-1α (red), GFAP, and NeuN in Cont, PT, and PT + IH groups. Confocal orthogonal views of the zoomed-in area (white inset) in the PT + IH group are shown. Quantitative analysis of HIF-1α intensity. n = 8**. (C)** Representative immunofluorescence images of Tom20 (green, mitochondrial marker) in Cont, PT, and PT + IH groups. Image processing workflow via Fiji (Analyze particles) to assess mitochondrial fragmentation, with classification into total, continuous, and small particles. Quantitative analysis of Tom20 intensity, total fragmentation, small fragmentation (<1.5 μm), and continuous fragmentation (>2 μm). Scale bar = 10 μm. n = 8**.** Data are presented as mean ± SEM. ns: not significant; ^*^P < 0.05 *vs.* Cont; *^#^P* < 0.05 *vs.* PT.

HIF-1α is an essential regulator that mediates adaptive responses to ischemia and hypoxia 46. We next assessed HIF-1α expression in the peri-infarct region to understand how IH exerts its neuroprotective effects. Quantitative Western blot analysis (**Figure 4a**) revealed that IH treatment significantly elevated HIF-1α levels in total protein extracts compared with the PT and control groups. In contrast, HIF-1α levels did not differ significantly between PT and control animals.

Furthermore, representative images of triple staining of NeuN/GFAP/HIF-1α showed that PT stroke animals presented a markedly increased astrocyte-specific HIF-1α compared to the control animals, and IH treatment further promotes the levels of HIF-1α (**Figure 4B**). HIF-1α is an emerging regulator for mitochondrial dynamics in response to oxidative stress and hypoxia 47, 48. Therefore, we next sought to explore the effects of IH on mitochondrial fragmentation. As shown in **Figure 4C**, the images of Tom20 fluorescent staining were processed and quantitatively analyzed. PT stroke animals displayed significantly decreased Tom20 intensity, along with an increased number of small fragmented particles (< 1.5 μm) and a reduced number of continuous mitochondrial networks (> 2 μm) compared to the control animals. Intriguingly, IH alleviates PT stroke-induced excessive mitochondrial fragmentation.

To further explore the molecular mechanisms underlying IH-mediated mitochondrial protection, we examined the expression of key regulators of mitochondrial fusion. As shown in **Figure S2A**, PT stroke significantly reduced the protein levels of MFN1, MFN2, and OPA1 compared to the control group, indicating impaired mitochondrial fusion capacity. In contrast, IH treatment effectively reversed these reductions, consistent with the improved mitochondrial morphology observed by Tom20 staining. Furthermore, to assess mitochondrial functional outcomes, we measured ATP levels in peri-infarct tissues using a luciferase-based assay. As presented in **Figure S2B**, PT stroke resulted in a marked decrease in ATP content, which was significantly restored by IH treatment. These findings collectively suggest that IH treatment not only preserves mitochondrial structural integrity but also enhances mitochondrial bioenergetic function, likely through the upregulation of fusion-related proteins and improved ATP production.

### IH treatment modulates glial phenotypes and reverses PT-induced cell death and inflammatory pathways

**Figure 5:**
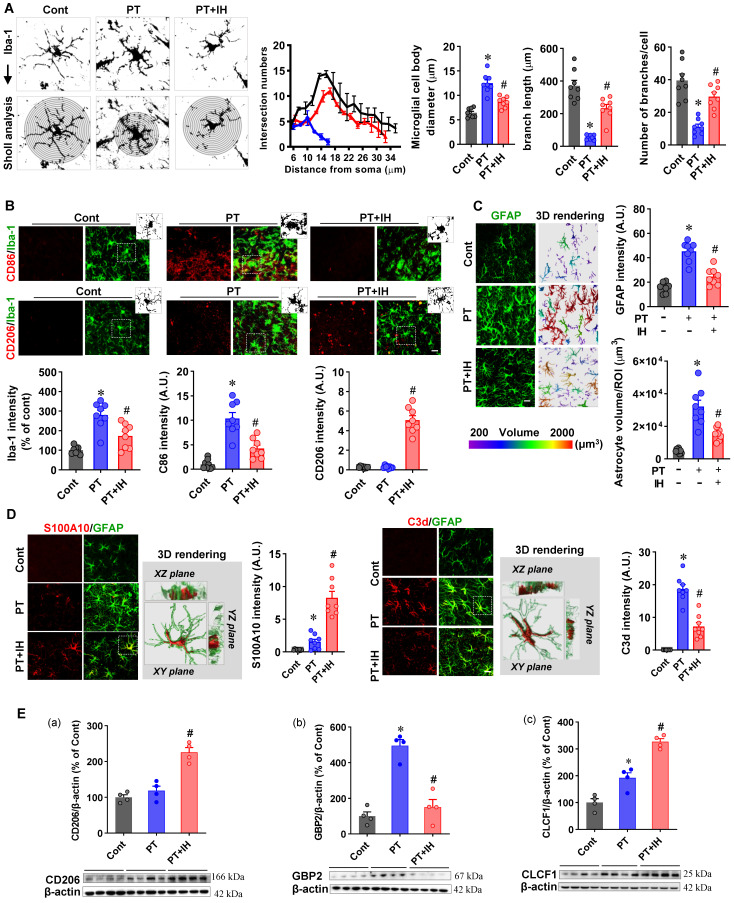
** IH attenuated the excessive activation of microglia and astrocytes. (A)** Representative images of Iba-1 (microglial marker) staining with Sholl analysis and quantification of microglial cell body diameter, branch length, and number of branches per cell. **(B)** Representative immunofluorescence images of Iba-1 with CD86 (an M1 microglial marker) and CD206 (an M2 microglial marker) co-staining. Quantitative analysis of Iba-1 intensity, CD86 intensity, and CD206 intensity. n = 8 per group; **(C)** Representative immunofluorescence images of GFAP with 3D rendering (pseudocolored by volume) and quantification of GFAP intensity and astrocyte volume per ROI. n = 8 per group;** (D)** Representative immunofluorescence images of S100A10/GFAP and C3d/GFAP co-staining. The confocal orthogonal view of the zoomed-in 3D rendering images in a typical PT group is magnified. Quantitative analysis of S100A10 intensity and C3d intensity. n = 8 per group; **(E)** Western blot images and quantitative analysis of CD206, GBP2, and CLCF1 protein expression, with β-actin as the loading control; n = 4 per group. Scale bar indicates 10 μm. All data are mean ± SEM. ns denotes no significance; *P < 0.05 compared with Cont; *^#^P* < 0.05 compared with PT.

Emerging evidence has established a link between HIF-1α and post-stroke inflammation, while excessive activation of microglia and astrocytes is a well-recognized pathological hallmark of PT stroke 9, 49. Building on our prior observation that IH promotes astrocyte-specific HIF-1α expression, we next investigated whether IH modulates the activation of microglia and astrocytes. To assess microglial activation, we first performed Sholl analysis to quantify microglial morphological changes (**Figure 5A**). As shown in **Figure 5A**, PT stroke caused a significant increase in microglial cell body diameter and a remarkable decrease in branch length, branch number, and intersection numbers with the concentric circles in Sholl analysis. However, these changes were effectively suppressed by IH treatment.

To further assess microglial activation, we performed double immunofluorescence for Iba-1 (a general microglial marker) together with CD86 (M1 pro-inflammatory) and CD206 (M2 anti-inflammatory; **Figure 5B**). PT stroke significantly elevated Iba-1 and CD86 signals, whereas IH treatment not only reduced these PT-induced increases but also upregulated CD206 expression (**Figure 5B**). Together with the observed morphological changes, these shifts in M1/M2 markers indicate that IH promotes microglial polarization from a pro-inflammatory (M1) toward an anti-inflammatory (M2) phenotype.

Similar changes were detected in astrocytes following PT stroke and IH treatment. As presented in **Figure 5C**, PT stroke resulted in significantly increased GFAP intensity, which was attenuated in the peri-infarct region following IH treatment. To further characterize astrocyte activation, we performed 3D rendering analysis of GFAP immunofluorescent images, which revealed that PT stroke significantly increased astrocyte volume. Consistent with its effect on GFAP intensity, IH treatment alleviated this stroke-induced increase in astrocyte volume.

In addition, we examined astrocyte phenotypic polarization by co-labeling GFAP with S100A10 (a neuroprotective A2 astrocyte marker) and C3d (a neurotoxic A1 astrocyte marker) in the peri-infarct region. As shown in **Figure 5D**, PT stroke caused a significant increase in C3d and S100A10 intensity, compared to the control animals. However, IH treatment attenuated the stroke-induced upregulation of C3d and further elevated S100A10 expression, indicating a remarkable shift of astrocytes from A1 phenotype to A2 phenotype.

To further confirm the effect of IH on microglial and astrocyte phenotypic polarization, we performed additional molecular analyses. As shown in **Figure 5E**, Western blot analysis revealed that IH treatment upregulated CD206 and CLCF1 (an A2 astrocyte marker), while downregulating GBP2 (an A1 astrocyte marker). Consistently, qPCR analysis (**Figure S3A-C**) showed that IH downregulated mRNA expression of the M1 marker *Cd86*, while upregulating that of the M2 microglial markers *Cd206* and *Arg1*. Furthermore, bulk RNA-seq transcriptomic analysis (**Figure S3D**) demonstrated that PT stroke upregulated pro-inflammatory markers, including *Cd86*, *Gbp2*, *Ccl3*, and *Cxcl13*, as well as A1 astrocyte markers such as *Serping1* and *H2-D1*. Conversely, analysis of the IH vs. PT comparison (**Figure S3E**) revealed that IH treatment upregulated genes associated with blood-brain barrier integrity (*Cldn5*), neuroprotection (*Vgf*), and neuronal modulation (*Kcnip3*), while downregulating the homeostatic microglial marker *Tmem119* and the NF-κB pathway inhibitor *Nfkbia*. Collectively, these transcriptional changes suggest a shift toward an anti-inflammatory and repair-supportive microenvironment, albeit with complex modulation of microglial homeostatic signatures that requires further investigation. To further characterize the biological pathways affected by IH, we performed GSEA of KEGG pathways using the same RNA-seq dataset. Compared with the PT group, IH significantly downregulated multiple stroke-enriched pathways, including cell death programs (Apoptosis, Ferroptosis, Necroptosis) and core inflammatory cascades (NF-κB, Toll-like receptor, JAK-STAT, TGF-β) (FDR q < 0.05, **Table S5**). These pathway-level findings indicate that IH broadly suppresses pro-death and pro-inflammatory signaling, complementing the gene-level anti-inflammatory and pro-repair changes observed in our earlier analyses.

### IH treatment promotes neurogenesis and angiogenesis in the peri-infarct following PT stroke

**Figure 6:**
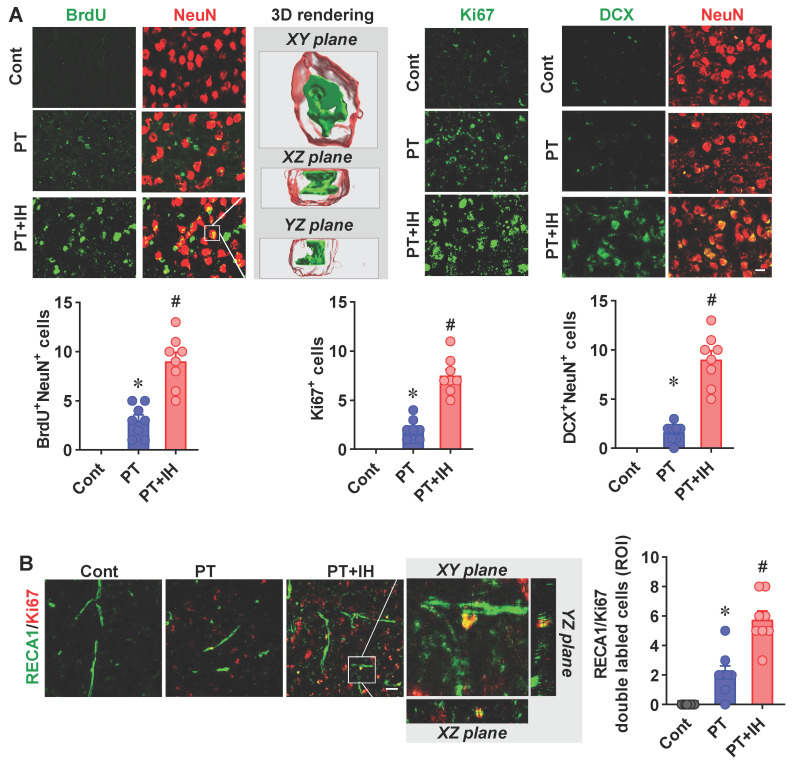
** IH treatment promoted neurogenesis and angiogenesis in the peri-infarct following PT stroke. (A)** Representative immunofluorescence images of BrdU/NeuN (left, with 3D rendering showing XY, XZ, and YZ planes of the zoomed area), Ki67 (middle), and DCX/NeuN (right) staining. Quantitative analysis of BrdU⁺/NeuN⁺ cells, Ki67⁺ cells, and DCX⁺/NeuN⁺ cells. (B) Representative immunofluorescence images of RECA1 (vascular marker) and Ki67 co-staining, with zoomed-in orthogonal views. Quantitative analysis of RECA1⁺/Ki67⁺ cells per ROI. Scale bar=10 μm. Data are presented as mean ± SEM. n = 8. ns: not significant; ^*^P < 0.05 *vs.* Cont; *^#^P* < 0.05 *vs.* PT.

To determine whether IH treatment affects neurogenesis and angiogenesis in the peri-infarct region following PT stroke, we performed immunofluorescence staining and quantitative analysis. Cell proliferation and neurogenesis were assessed using BrdU (a proliferation marker), Ki67, and DCX (a neuroblast marker) (**Figure 6A**). Compared with the control group, the PT stroke group showed a significant increase in the number of BrdU⁺, Ki67⁺, and DCX⁺ cells, indicating an endogenous stroke-induced proliferative response. Notably, IH treatment further significantly increased these counts relative to the PT group, suggesting that IH amplifies post-stroke neurogenesis.

Furthermore, angiogenesis was evaluated via co-staining for RECA1 (a vascular endothelial marker) and Ki67 (**Figure 6B**). The PT stroke group exhibited a significantly higher number of RECA1⁺/Ki67⁺ proliferative endothelial cells than the control group, reflecting basal post-stroke angiogenesis. IH treatment further augmented this angiogenic response, with a significantly greater number of double-labeled cells observed in the PT+IH group compared with the PT group.

## Discussion

Ischemic stroke remains a major global health burden and a key cause of disability and death globally, with elderly populations facing disproportionately higher risks of incidence and poorer post-stroke recovery outcomes 50. The limited efficacy and strict contraindications of current approved therapeutic approaches in older patients underscore an urgent need to seek safer, more accessible interventions 4, 5. In the present study, we investigated the effects of IH post-conditioning on brain injury and functional deficits induced by ischemic stroke. Using a PT stroke model in 25-month-old mice, our findings demonstrated that IH treatment, consisting of seven total sessions administered on days 3 to 16 post-stroke and with each session comprising five cycles of 10-minute exposure to 8% oxygen, confers robust neuroprotective effects in aged stroke-affected mice. Specifically, we observed that IH induces a marked reduction in cortical infarct volume, while also ameliorating sensorimotor deficits and alleviating neuronal damage and apoptosis. Further investigations revealed that IH preserves vascular morphology, attenuates excessive astrocyte-vasculature interactions, mitigates the excessive activation of microglia and astrocytes, and promotes the anti-inflammatory polarization of glial cells. Importantly, we also noted a significant increase in astrocyte-specific HIF-1α expression, as well as the preservation of mitochondrial integrity. Collectively, these multifaceted changes act in concert to promote neurogenesis and angiogenesis.

Our findings demonstrate that IH post-conditioning confers neurorestoration in aged stroke mice, a result that aligns with a growing body of literature exploring the therapeutic potential of modified respiratory gases 51-53. A significant body of previous work has extensively investigated the neuroprotective effects of hypercapnic hypoxia, the combination of elevated CO₂ and low O₂ 51-55. These foundational studies demonstrated that the combination of hypercapnia and hypoxia exerts a more pronounced neuroprotective effect than either stimulus alone, significantly reducing brain damage in rodent ischemia models 54, 55. Mechanistically, this potentiation is linked to enhanced HIF-1α activation, anti-apoptotic signaling, and the modulation of mitochondrial function and glial cells 51-55. Importantly, this approach has recently been translated into clinical settings, showing promising results in the rehabilitation of patients after ischemic stroke 51.

While this previous research powerfully establishes the efficacy of hypercapnic hypoxia 51-55, it is crucial to distinguish our work from these studies. The IH protocol used in our current study involves normocapnic hypoxia (8% O₂ without supplemental CO₂), a distinct physiological intervention from hypercapnic hypoxia. The central and novel contribution of our work is the demonstration that even IH alone is a potent neuroprotective strategy in a clinically relevant aged (25-month-old) mouse model of stroke. Furthermore, we provide a comprehensive and detailed mechanistic exploration in this aged context, revealing that IH’s benefits are mediated through a multi-faceted process involving astrocyte-specific HIF-1α upregulation, preservation of mitochondrial integrity, and a shift in glial cell polarization towards anti-inflammatory (A2/M2) phenotypes. By situating our findings within the context of the hypercapnic hypoxia literature, we not only acknowledge the significant advances in the field but also clarify that our work with IH in an aged model offers a distinct and translationally relevant avenue for stroke therapy.

The primary clinical goal of post-stroke therapy is to limit infarct expansion and alleviate the deficits in sensorimotor function 9, 56. However, impaired brain repair capacity in aged individuals exacerbated the brain injury and behavioral deficits following ischemic stroke 2, 57. In our study, IH treatment significantly reduced cortical infarct volume in PT-stroke aged mice, a finding consistent with prior observations in young middle cerebral artery occlusion models 21. In addition, our studies revealed a significant amelioration in PT-induced deficits across a comprehensive battery of behavioral tests, a result of particular note given that aged mice typically exhibit delayed and substantially less complete functional recovery post-stroke relative to their young counterparts 58. Neuronal damage, apoptosis, and synaptic injury are major drivers of post-stroke disability 9, 59. Consistent with our previous study, immunofluorescence analyses in the current study revealed that PT stroke drastically increased the activation of apoptotic cascades and reduced the number of surviving neurons in the peri-infarct cortex of aged mice 9. In contrast, IH treatment attenuates these effects, significantly decreasing cleaved-caspase-3/9 intensity, alleviating neuronal loss and synaptic damage. These findings underscore IH’s potential to address a critical unmet clinical need in elderly stroke patients.

Cerebrovascular damage and impaired vascular repair are typical pathophysiological features of ischemic stroke, and age-related vascular dysfunction further worsens outcomes 60, 61. A previous clinical study found that BBB disruption following acute ischemic stroke predicts functional outcome, which highlights the BBB’s role as a key prognostic indicator beyond the traditional metric of stroke size 61. Pre-clinical studies revealed that PT stroke induced BBB disruption and morphological changes, and prolonged BBB disruption was closely associated with neuroinflammatory response 3, 62, 63. Our current study found that PT stroke significantly reduced vascular density, surface area, length density, and capillary volume in the peri-infarct region. Intriguingly, IH treatment reversed these morphological deficits. This finding is critical because normal vessel morphology and intact vasculature are required to deliver oxygen and nutrients to the peri-infarct area, supporting neuronal survival and repair 3. Notably, we also observed excessive astrocyte-vasculature interactions in PT-stroke mice. While extensive astrocyte-vascular interactions are indispensable for maintaining the integrity of the BBB, excessive engagement between astrocytes and the vasculature can undermine both vascular remodeling and BBB integrity 3, 64. Interestingly, our study found that IH treatment could attenuate excessive astrocyte-vascular interactions, which contribute to preserving BBB function and facilitating vascular repair.

The therapeutic potential of hypoxia treatment has attracted growing interest across multiple medical fields 16. HIF-1α is a widely studied oxygen-sensitive transcription factor in response to hypoxia 65, 66. In the current study, we analyzed total HIF-1α expression in the peri-infarct cortex via Western blot, using β-actin as a loading control. This approach was chosen to assess overall changes in HIF-1α abundance, rather than to precisely quantify nuclear translocation efficiency. Using this method, we found that IH markedly increased total HIF-1α levels in the peri-infarct cortex. Immunofluorescence staining further demonstrated that this upregulation was predominantly restricted to astrocytes, with minimal induction in neurons. While nuclear translocation is essential for HIF-1α transcriptional activity, quantitative assessment of nuclear/cytoplasmic distribution in reactive astrocytes of the ischemic penumbra is technically challenging due to their irregular nuclear morphology and dense cytoplasmic processes. Therefore, the present study prioritized qualitative confirmation of cell-type-specific HIF-1α induction, which sufficiently supports our core findings. Future studies will employ adult astrocyte isolation combined with nuclear-cytoplasmic fractionation to precisely quantify HIF-1α nuclear translocation and further validate its functional activation in astrocytes.

The findings on HIF-1α following IH are consistent with HIF-1α expression patterns in neurons and astrocytes following ischemic insults 67. Specifically, neuronal HIF-1α exhibits a rapid yet transient response to ischemic stress, whereas astrocytic HIF-1α responds with a delayed onset and sustained upregulation 67. This cell-type-specific HIF-1α change aligns with the functional role of astrocytes in post-stroke tissue repair 68. A previous study has demonstrated that the elevated astrocytic HIF-1α levels contributed to the improved neurological recovery in ischemic stroke by enhancing angiogenesis 68. Our data extend this insight by showing that IH can recapitulate this beneficial astrocyte-specific HIF-1α and promote angiogenesis in the peri-infarct cortex, suggesting that astrocytes may serve as long-term hypoxia “regulatory hubs” in the peri-infarct area, coordinating with repair processes. Moreover, the beneficial role of HIF-1α is not limited to promoting angiogenesis, as accumulating evidence has demonstrated that it has recently emerged as a key regulator of mitochondrial dynamics and respiratory function 69. Consistent with our transcriptomic pathway analysis, IH reversed PT-induced enrichment of cell death pathways (Apoptosis, Ferroptosis, Necroptosis) and inflammatory pathways (NF-κB, Toll-like receptor, JAK-STAT). These data provide a pathway-level molecular link between IH-induced astrocytic HIF-1α upregulation and the observed multi-cellular protection, including reduced neuronal apoptosis, anti-inflammatory glial polarization, and preserved vascular integrity. At the subcellular level, our data show that PT stroke caused significant mitochondrial fragmentation, as evidenced by reduced Tom20 intensity, increased total particle count, and more small, fragmented mitochondria. IH treatment reversed these changes, preserving mitochondrial integrity. These findings suggest a possible mechanistic cascade in which the IH-induced increased HIF-1α preserves mitochondrial integrity and suppresses mitochondria-mediated apoptosis.

Additionally, our study found that IH post-conditioning also exerts a pivotal role in modulating the phenotypic polarization of glial cells. Following an acute stroke, neuroinflammation exerts dual roles, either exacerbating brain injury by releasing pro-inflammatory factors or conversely playing a beneficial role by promoting recovery 9, 39. The specific effect of neuroinflammation depends on the duration of the inflammatory response and the polarization status of glial cells. In the current study, PT stroke-induced excessive activation of microglia and astrocytes lasted until day 20 post-stroke, with microglia and astrocytes mainly displaying A1/M1 pro-inflammatory phenotype. IH treatment, however, markedly suppressed these alterations and drove glial polarization from a pro-inflammatory (A1/M1) toward an anti-inflammatory (A2/M2) phenotype. In contrast, a prior study reported that elevated HIF-1α in the peri-infarct region at 24 h after injury triggered robust microglial and astrocytic activation and worsened neuroinflammation 70. Our research further demonstrates that increased astrocytic HIF-1α expression at 20 days post-stroke alleviates neuroinflammation by promoting the anti-inflammatory polarization of glial cells. This observation establishes the regulatory role of HIF-1α in microglia and astrocytes throughout the time course of ischemic stroke. It should be noted that the previous study did not specify the cellular type in which HIF-1α was expressed 70. However, existing research has shown that neuronal HIF-1α exhibits a rapid yet transient response to ischemic stress, while astrocytic HIF-1α responds with a delayed onset and sustained upregulation 67. Based on this evidence, we speculate that neuronal HIF-1α may contribute to the promotion of neuroinflammation in the early post-stroke phase, whereas astrocytic HIF-1α plays an anti-inflammatory role during the long-term period following IH post-conditioning, although more studies in the future are still needed. Finally, our findings confirmed that IH treatment could enhance neurogenesis. This beneficial effect may be attributed to multiple mechanisms, including the preservation of vascular morphology, the attenuation of excessive astrocyte-vasculature interactions, the enhancement of mitochondrial dynamics, and the regulation of glial cell phenotypes.

We also confirmed the safety and tolerability of the intermittent hypoxia (IH) protocol in 25-month-old mice subjected to photothrombotic (PT) stroke. No significant differences in body weight were observed across groups at the experimental endpoint, and the PT+IH group exhibited a higher 20-day survival rate compared with the PT and Cont groups (**Figure S4**). Notably, the single mortality in the PT+IH group occurred before the initiation of IH intervention, with no additional deaths or adverse reactions observed during the entire treatment period. Collectively, these findings demonstrate that the applied IH regimen is well-tolerated in aged stroke mice, providing a critical foundation for evaluating its therapeutic efficacy. To establish causality, future work should prioritize functional validation via astrocyte-specific HIF-1α manipulation, aiming to further elucidate the specific molecular pathways and cell-to-cell crosstalk governed by astrocytic HIF-1α in IH-mediated neuroprotection.

## Conclusions

**Figure 7:**
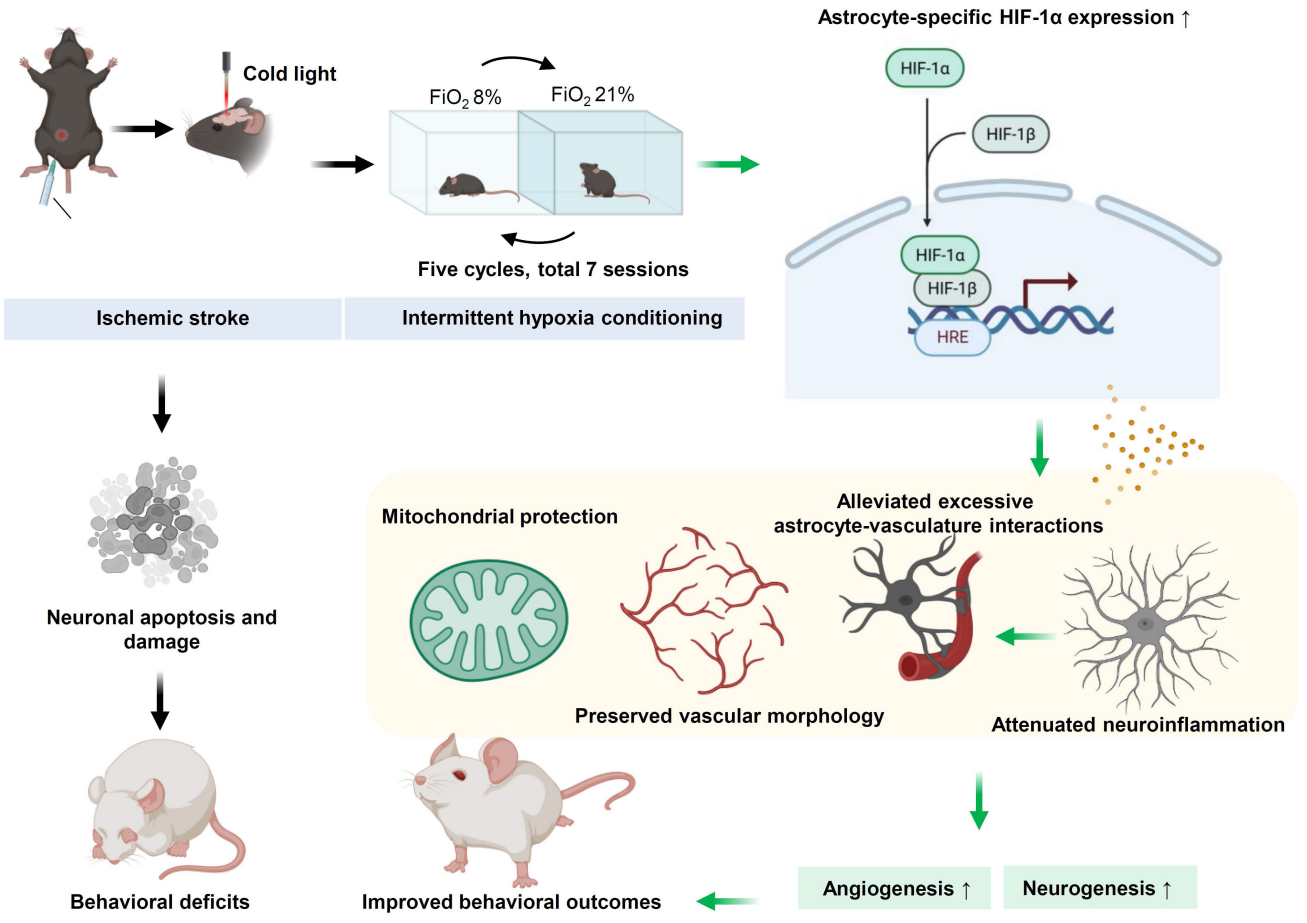
** Possible neuroprotective mechanism of IH in aged mice with PT stroke.** Photothrombotic stroke induces neuronal apoptosis, damage, and behavioral deficits in aged mice. IH treatment, via upregulating astrocyte-specific HIF-1α that binds to HREs, preserves mitochondrial and vascular morphology, alleviates astrocyte -vasculature interactions and neuroinflammation, and promotes angiogenesis and neurogenesis, ultimately exerting neurorestoration and improving behavioral outcomes.

Collectively, our study demonstrates that IH post-conditioning exerts robust neuroprotective effects in aged mice with PT stroke, including reducing cortical infarct volume, ameliorating sensorimotor deficits, alleviating neuronal apoptosis and synaptic damage, and enhancing neurogenesis and angiogenesis. These benefits are driven by coordinated mechanisms, including preserving cerebrovascular morphology and blood-brain barrier integrity, promoting anti-inflammatory polarization of microglia/astrocytes, upregulating astrocyte-specific HIF-1α, and maintaining mitochondrial integrity (**Figure 7**). By targeting age-exacerbated stroke pathologies, IH may offer a promising therapeutic strategy for elderly stroke care. While the safety of 8% FiO₂ exposure in aged humans requires further investigation, a recent clinical trial demonstrated that hypercapnic hypoxia (15-16% FiO₂) improved outcomes in stroke patients 51, supporting the therapeutic potential of modified respiratory gas interventions. Further studies are needed to optimize IH parameters and establish its safety profile before clinical translation.

## Supplementary Material

Supplementary figures and tables.

## Data Availability

All data generated or analyzed during this study are included in this published article.

## Abbreviations

tPA: tissue plasminogen activator
CBF: cerebral blood flow
HIF-1α: Hypoxia-inducible factor-1α
IH: Intermittent hypoxia
PT: photothrombotic
ip: intraperitoneal
PVDF: polyvinylidene difluoride
BSA: bovine serum albumin
HRP: horseradish peroxidase
ECL: enhanced chemiluminescence
ANOVA: one-way analysis of variance
SEM: standard error of the mean
