# Supplementary material for: Intermittent hypoxia ameliorates behavioral deficits and exerts neurorestoration in a mouse photothrombotic stroke model
Source: Theranostics. 2026 May 29;16(13):7160–77. doi: 10.7150/thno.126126 (PMC13294984; doi:10.7150/thno.126126)
Supplement: Supplementary file 1 — Supplementary figures and tables. [file thnov16p7160s1.pdf]

Supplementary Figures

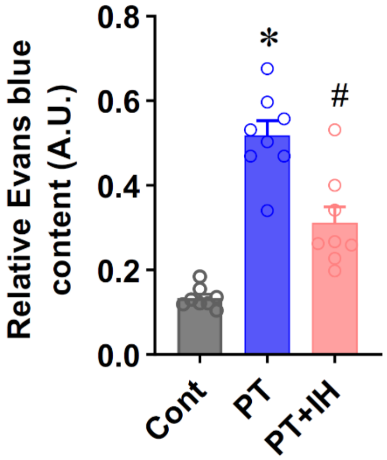

Supplementary Figure 1. IH treatment attenuates Evans blue extravasation in PT stroke mice. Relative Evans blue content in the ipsilateral cerebral cortex of mice in the Cont, PT, and PT+IH groups. Data are presented as mean ± SEM. n=8. *P < 0.05 *vs*. Cont group; $^{\#}P$ < 0.05 *vs*. PT group. A.U., arbitrary units.

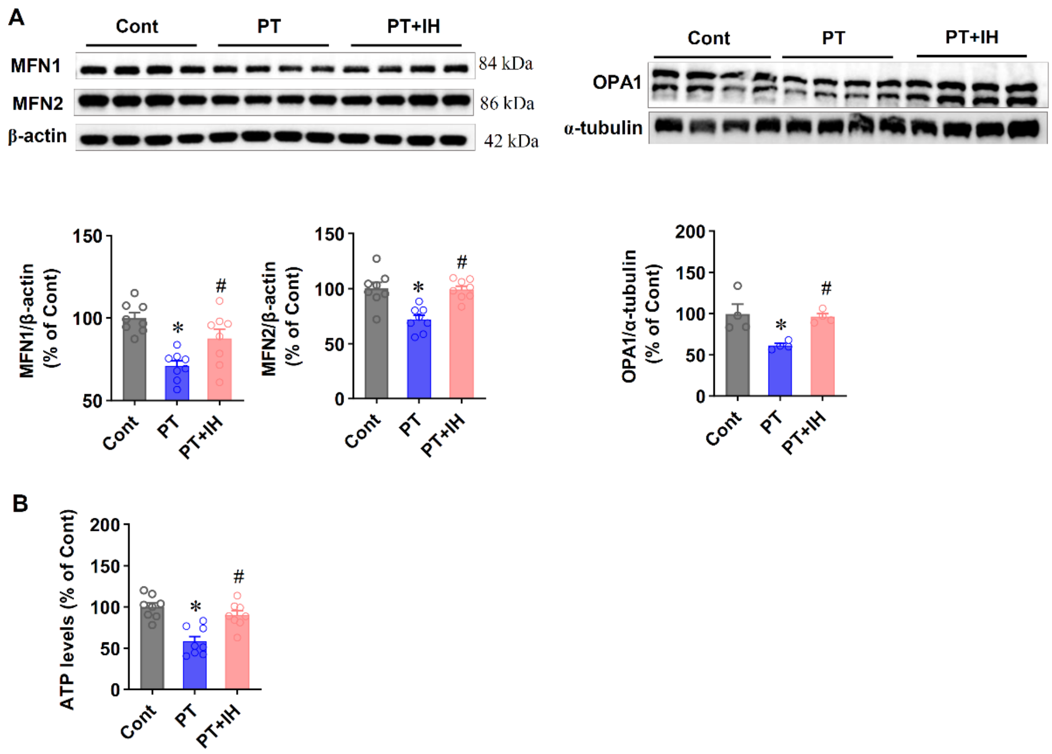

Supplementary Figure 2. IH treatment restores mitochondrial dynamics and ATP production in PT stroke mice. (A) Representative Western blot images and quantitative analysis of mitochondrial fusion proteins MFN1, MFN2, and OPA1 in the peri-infarct area. β-actin and α-tubulin were used as internal loading controls. (B) Quantitative analysis of ATP levels in the peri-infarct area of mice across the three groups. All data are normalized to the Cont group and presented as mean ± SEM. $^{*}P < 0.05$ *vs.* Cont group; $^{\#}P < 0.05$ *vs.* PT group.

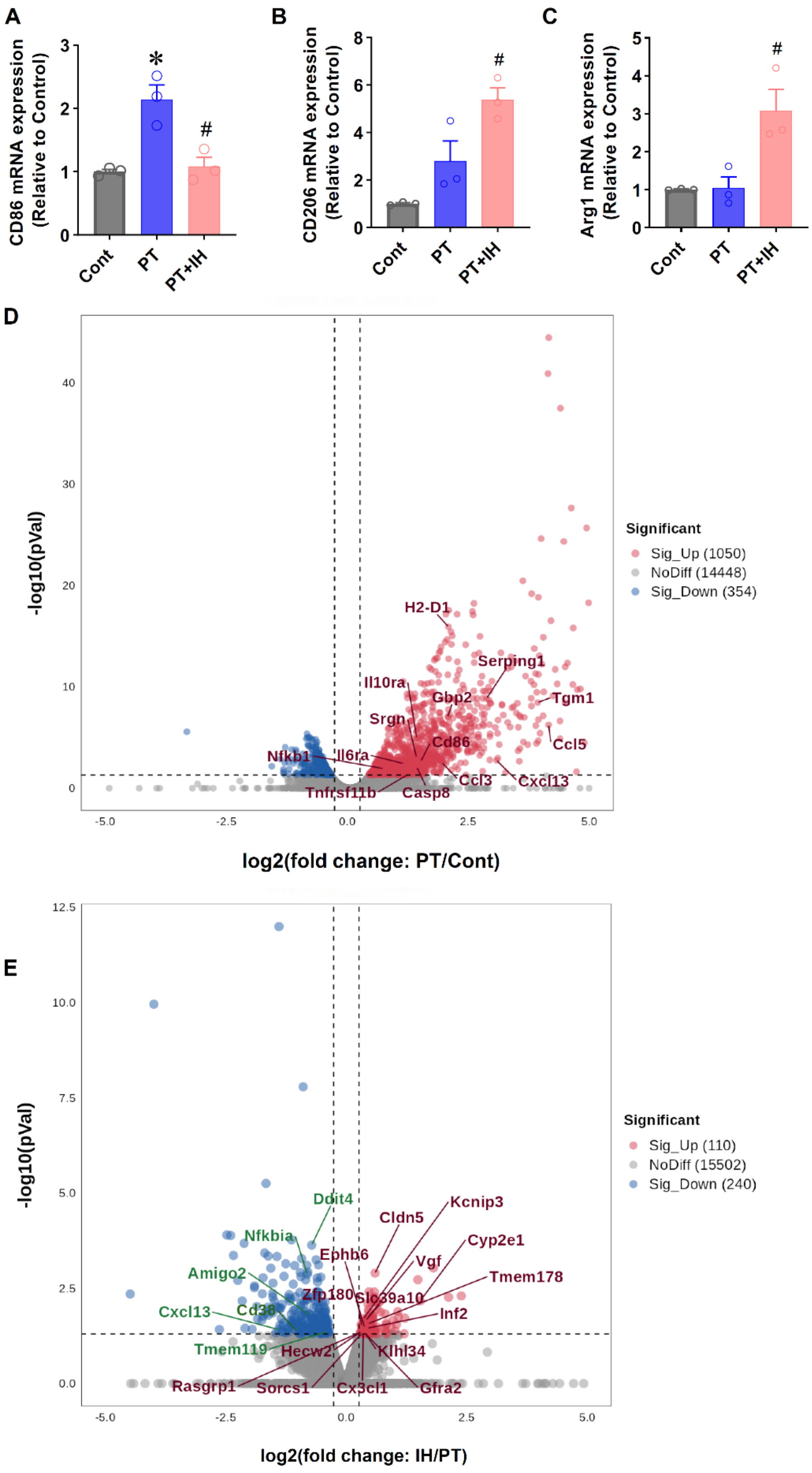

Supplementary Figure 3. IH treatment modulates microglial polarization and transcriptomic profile in PT stroke mice. (A-C) RT-qPCR analysis of CD86, CD206, and Arg1 mRNA expression in the peri-infarct area of mice in the Cont, PT, and PT+IH groups, normalized to the Cont group. (D) Volcano plot showing

differentially expressed genes (DEGs) between the PT and Cont groups, with red dots indicating significantly upregulated genes, blue dots indicating significantly downregulated genes, and gray dots indicating no significant difference (NoDiff, 14448). Key pro-inflammatory and microglial activation-related genes (e.g., Cd86, Il6ra, Nfkb1) are labeled. (E) Volcano plot showing DEGs between the PT+IH and PT groups, with red dots indicating significantly upregulated genes, blue dots indicating significantly downregulated genes, and gray dots indicating no significant difference. Key genes involved in inflammation, barrier function, and metabolism are labeled. All RT-qPCR data are presented as mean ± SEM. n=3. $^*P < 0.05$ *vs.* Cont group; $^\#P < 0.05$ *vs.* PT group.

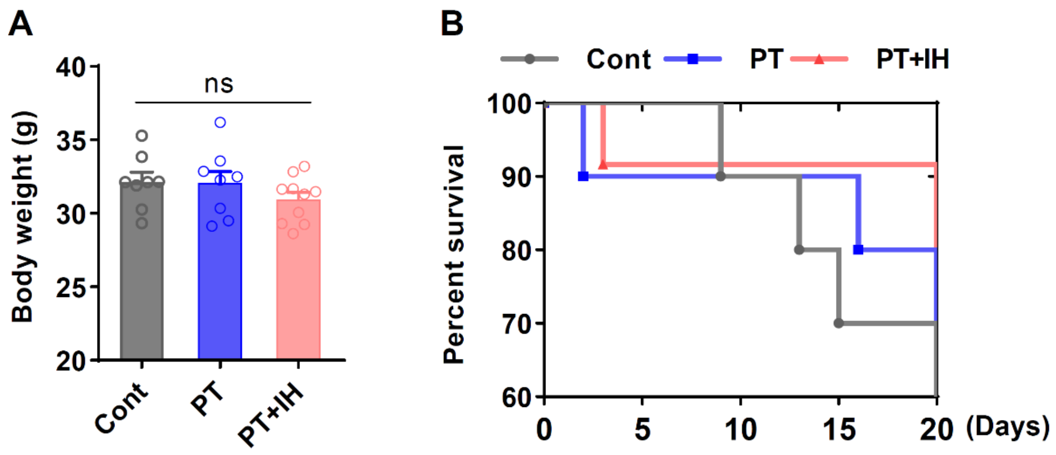

Supplementary Figure 4. IH treatment does not affect body weight and improves long-term survival in PT stroke mice. (A) Body weight of mice in the Cont, PT, and PT+IH groups at the endpoint of the experiment. No significant difference (ns) was observed among the three groups. Data are presented as mean ± SEM. n=8-10. (B) Kaplan-Meier survival curve showing the percent survival of mice in the three groups over a 20-day observation period following PT stroke induction.

Fig. S1

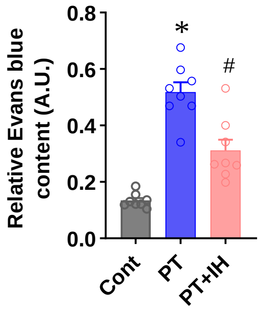

Fig. S2

A

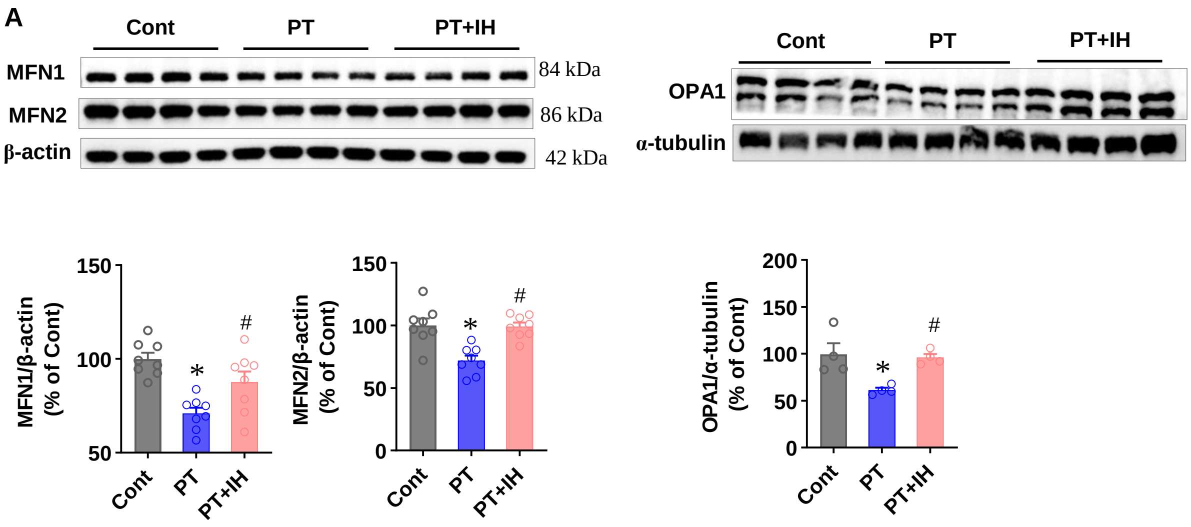
Cont
PT
PT+IH
MFN1
MFN2
β-actin
84 kDa
86 kDa
42 kDa
OPA1
α-tubulin
MFN1/β-actin (% of Cont)
MFN2/β-actin (% of Cont)
OPA1/α-tubulin (% of Cont)
150
100
50
0
200
*
#

B

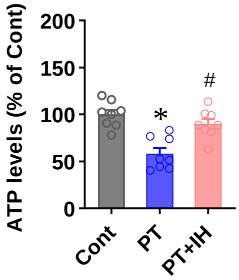
ATP levels (% of Cont)
200
150
100
50
0
*
#
Cont
PT
PT+IH

Fig. S3

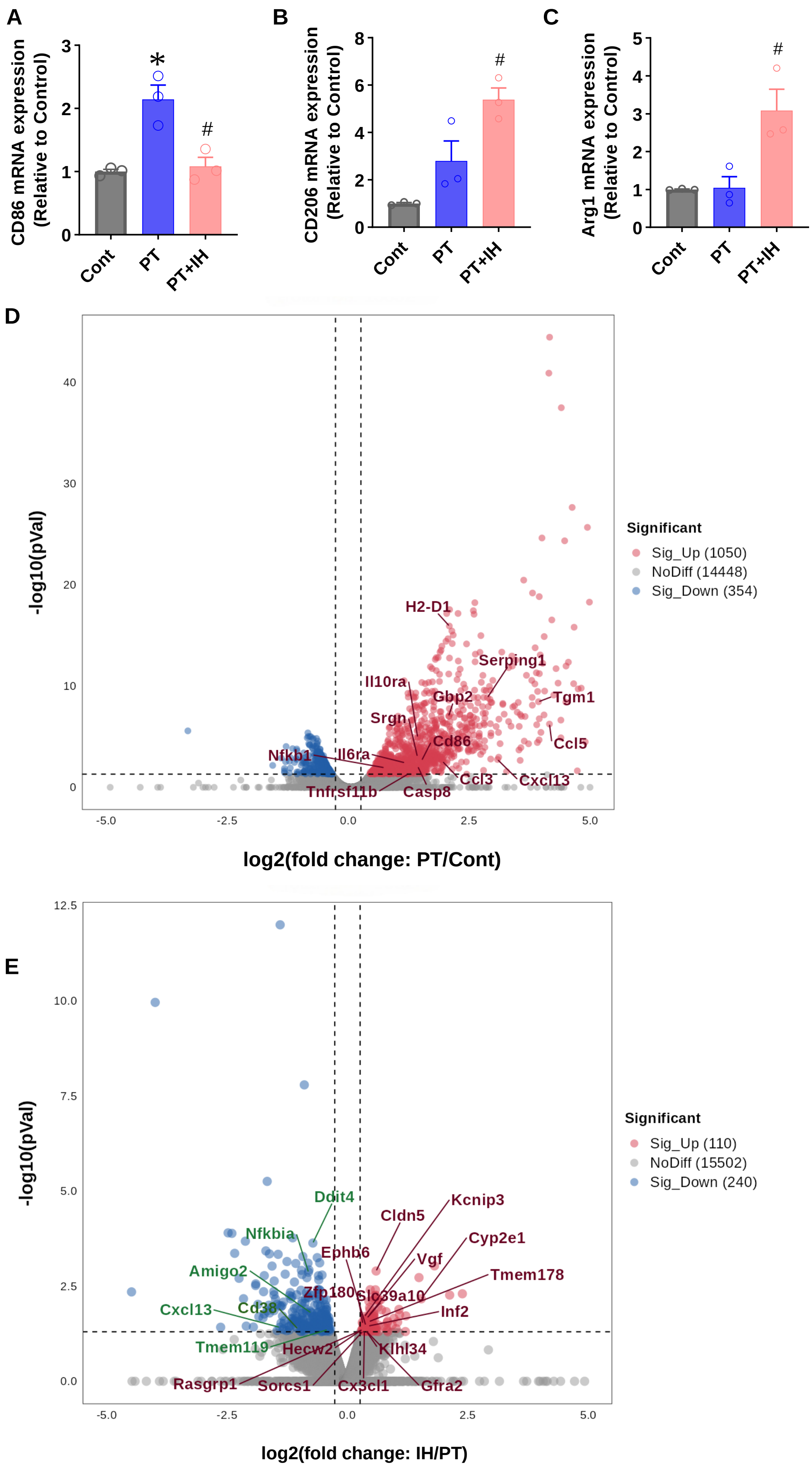

A
CD86 mRNA expression (Relative to Control)
Cont
PT
PT+IH
B
CD206 mRNA expression (Relative to Control)
C
Arg1 mRNA expression (Relative to Control)
D
-log10(pVal)
log2(fold change: PT/Cont)
Significant
Sig_Up (1050)
NoDiff (14448)
Sig_Down (354)
H2-D1
Serping1
Il10ra
Gbp2
Tgm1
Srgn
Cd86
Ccl5
Nfkb1
Il6ra
Ccl3
Cxcl13
Tnfrsf11b
Casp8
E
-log10(pVal)
log2(fold change: IH/PT)
Significant
Sig_Up (110)
NoDiff (15502)
Sig_Down (240)
Ddit4
Kcnip3
Cldn5
Cyp2e1
Nfkbia
Ephb6
Vgf
Amigo2
Tmem178
Zfp180
Slc39a10
Cxcl13
Cd38
Inf2
Tmem119
Hecw2
Klhl34
Rasgrp1
Sorcs1
Cx3cl1
Gfra2

Fig. S4

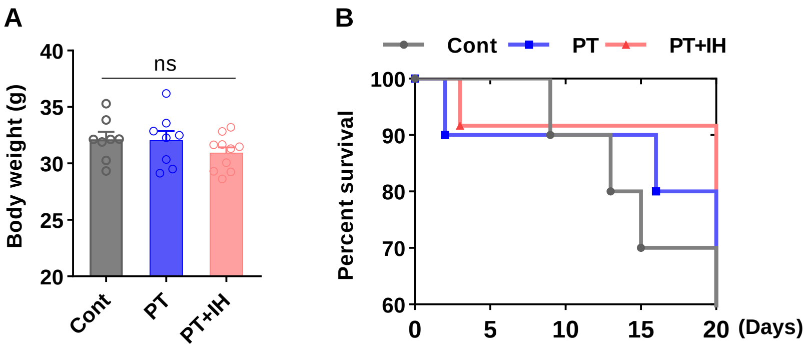
A
B
Body weight (g)
40
35
30
25
20
ns
Cont
PT
PT+IH
Cont
PT
PT+IH
Percent survival
100
90
80
70
60
0
5
10
15
20
(Days)

Supplementary Table 1. Antibodies used in this study

Antibody	Host	Catalog number	Vendor
NeuN	Rabbit	ET1602-12	HUABIO
Cleaved-caspase 3	Mouse	ARG66888	Arigobio
MAP2	Chicken	ab5392	Abcam
MBP	Rabbit	ab218011	Abcam
Synaptophysin	Mouse	S5768	Sigma
Spinophilin	Rabbit	#14136	CST
RECA1	Mouse	MCA970R	Bio-Rad
GFAP	Chicken	ab4674	Abcam
HIF-1α	Rat	HA721997	HUABIO
Tom20	Rabbit	11802	Proteintech
Iba-1	Rabbit	019-19741	FUJIFILM
CD86	Mouse	ab213044	Abcam
CD206	Rabbit	HA722892	HUABIO
S100A10	Rabbit	PA5-120679	Thermo Fisher
C3d	Goat	AF2655	R&D Systems
BrdU	Mouse	MA3-071	Thermo Fisher
DCX	Rabbit	48-1200	Thermo Fisher
Ki67	Rabbit	ab16667	Abcam
GBP2	Rabbit	DF2499	Affinity Biosciences
CLCF1	Rabbit	ABIN4902021	Antibodies-online
β-actin	Rabbit	4967	CST
MFN1	Rabbit	13798-1-AP	Proteintech
MFN2	Rabbit	12186-1-AP	Proteintech
OPA1	Rabbit	80471S	CST
α-Tubulin	Rabbit	2144S	CST

Supplementary Table 2. Primer sequences used for RT-qPCR.

Gene	Forward primer (5'→3')	Reverse primer (5'→3')
18S rRNA	CCCCTCGATGCTCTTAGCTG	GAACCGCGGTCCTATTCCAT
GAPDH	GCCTCCTCCAATTCAACCCTT	TGTCTACGGGACGAGGAAAC
CD86	CTGGACTCTACGACTTCACAATG	AGTTGGCGATCACTGACAGTT
CD206	CTCTGTTCAGCTATTGGACGC	TGGCACTCCCAAACATAATTTGA
Arg1	TGGCTTGCGAGACGTAGAC	GCTCAGGTGAATCGGCCTTT
TNFα	CCTGTAGCCCACGTCGTAG	GGGAGTAGACAAGGTACAACCC

Supplementary Table 3. Holm-Šídák multiple comparisons test results for all outcomes in aged PT stroke mice with IH treatment

Outcome	Comparison	Effect Size (Mean Diff.)	95% CI of Effect Size	Adjusted P Value
Infarct volume	PT vs.Cont	98.28	[76.57, 119.99]	<0.0001
	PT+IH vs. PT	-58.93	[-80.64, -37.22]	<0.0001
Grip strength test	PT vs.Cont	-0.0108	[-0.0174, -0.0042]	0.0049
	PT+IH vs. PT	0.0122	[0.0055, 0.0188]	0.0024
Beam Balance test (Score)	PT vs.Cont	-2.467	[-3.154, -1.780]	<0.0001
	PT+IH vs. PT	2.121	[1.449,2.793]	<0.0001
Beam Balance test (Average Speed)	PT vs.Cont	-0.0257	[-0.0401, -0.0114]	0.0021
	PT+IH vs. PT	0.0272	[0.0136, 0.0409]	0.0012
Relative contralateral paw use (%)	PT vs.Cont	-29.03	[-40.25, -17.81]	<0.0001
	PT+IH vs. PT	21.69	[9.99, 33.39]	0.0016
Removal time (Sec)	PT vs.Cont	107.7	[66.56, 148.84]	<0.0001
	PT+IH vs. PT	-84.86	[-127, -42.72]	0.0011
No. of foot slips/total steps (% of cont)	PT vs.Cont	399.5	[324.37, 474.63]	<0.0001
	PT+IH vs. PT	-342.6	[-419.42, -265.78]	<0.0001
Open field test Velocity (m/s)	PT vs.Cont	-0.0214	[-0.0344, -0.0084]	0.0046
	PT+IH vs. PT	0.026	[0.0127, 0.0394]	0.0013
Open field test Line crossings	PT vs.Cont	-68.91	[-109.68, -28.14]	0.0041
	PT+IH vs. PT	71	[30.23, 117.77]	0.0041
Cle-caspase 3 intensity (A.U.)	PT vs.Cont	6.191	[4.49, 7.90]	<0.0001
	PT+IH vs. PT	-3.978	[-5.68, -2.27]	0.0002
Cle-caspase 9 intensity (A.U.)	PT vs.Cont	7.587	[6.01, 9.16]	<0.0001
	PT+IH vs. PT	-4.315	[-5.89, -2.74]	<0.0001
Surviving neurons/ROI	PT vs.Cont	-23.38	[-31.05, -15.71]	<0.0001
	PT+IH vs. PT	11.88	[4.21, 19.55]	0.0081
MAP2 intensity (% of Cont)	PT vs.Cont	-32.26	[-48.06, -16.46]	0.0011
	PT+IH vs. PT	26.39	[10.59, 42.19]	0.0045
Area of continuous Structure (% of Cont)	PT vs.Cont	-26.46	[-42.74, -10.18]	0.0084
	PT+IH vs. PT	20.05	[3.77, 36.33]	0.036
Number of small Particles (ROI)	PT vs.Cont	1282	[459.7, 2104.3]	0.0117
	PT+IH vs. PT	-960.8	[-1781.1, -138.5]	0.0479
MBP intensity (% of Cont)	PT vs.Cont	-37.26	[-52.53, -21.99]	0.0002
	PT+IH vs. PT	17.36	[2.09, 32.63]	0.0278
Dispersion (% of Cont)	PT vs.Cont	25.66	[10.36, 40.96]	0.0067
	PT+IH vs. PT	-19.15	[-34.45, -3.85]	0.0332
SYP intensity (% of Cont)	PT vs.Cont	-38.56	[-50.17, -26.95]	<0.0001
	PT+IH vs. PT	18.3	[6.69, 29.91]	0.0036
Spin intensity (% of Cont)	PT vs.Cont	-28.5	[-40.73, -16.27]	0.0003
	PT+IH vs. PT	15.79	[3.56, 28.02]	0.0276
Colocalized puncta	PT vs.Cont	-41.67	[-58.01, -25.33]	<0.0001

(% of Cont)	PT+IH vs. PT	17.11	[0.77, 33.45]	0.041
Vascular density (% area)	PT vs.Cont	-7.213	[-8.91, -5.51]	<0.0001
	PT+IH vs. PT	3.613	[1.91, 5.31]	0.0005
Vascular surface (μm^2)	PT vs.Cont	-33085	[-38976, -27194]	<0.0001
	PT+IH vs. PT	22003	[16112, 27894]	<0.0001
Vascular length density (% area)	PT vs.Cont	-6.15	[-8.69, -3.61]	0.0002
	PT+IH vs. PT	2.675	[0.13, 5.22]	0.0399
Capillary Vascular volume (% area)	PT vs.Cont	-4.825	[-6.66, -2.99]	<0.0001
	PT+IH vs. PT	2.013	[0.18, 3.85]	0.0328
Vascular astrocytes volume (% of Cont)	PT vs.Cont	702.9	[595.7, 810.1]	<0.0001
	PT+IH vs. PT	-515.1	[-622.3, -407.9]	<0.0001
Vessel-associated astrocytes (% astrocytic volume)	PT vs.Cont	9.492	[6.92, 12.06]	<0.0001
	PT+IH vs. PT	-4.725	[-7.29, -2.16]	0.0018
HIF-1α intensity (A.U.) (IF)	PT vs.Cont	12.89	[8.83, 16.95]	<0.0001
	PT+IH vs. PT	9.065	[5, 13.13]	0.0001
Tom20 intensity (% of Cont)	PT vs.Cont	-33.4	[-46.86, -19.94]	0.0001
	PT+IH vs. PT	21.51	[8.05, 34.97]	0.0064
Total fragmentation	PT vs.Cont	0.5256	[0.369, 0.682]	<0.0001
	PT+IH vs. PT	-0.3044	[-0.461, -0.148]	0.0012
Small fragmentation (<1.5 μm)	PT vs.Cont	1.586	[1.066, 2.106]	<0.0001
	PT+IH vs. PT	-0.9520	[-1.472, -0.432]	0.0021
Continuous fragmentation (2 μm to infinity)	PT vs.Cont	0.13	[0.0912, 0.1688]	<0.0001
	PT+IH vs. PT	-0.07986	[-0.1187, -0.0410]	0.0007
Microglial cell body diameter (μm)	PT vs.Cont	6.071	[4.61, 7.53]	<0.0001
	PT+IH vs. PT	-3.828	[-5.29, -2.37]	<0.0001
Branch length (μm)	PT vs.Cont	-316.9	[-385.6, -248.2]	<0.0001
	PT+IH vs. PT	179.7	[111.0, 248.4]	<0.0001
Number of branches /cell	PT vs.Cont	-28.38	[-36.99, -19.77]	<0.0001
	PT+IH vs. PT	18.50	[9.89, 27.11]	0.0004
GFAP intensity (A.U.)	PT vs.Cont	29.18	[22.34, 36.02]	<0.0001
	PT+IH vs. PT	-20.66	[-27.50, -13.82]	<0.0001
Astrocyte volume/ROI (μm3)	PT vs.Cont	27095	[19786, 34404]	<0.0001
	PT+IH vs. PT	-16635	[-23944, -9326]	0.0002
Iba-1 intensity (% of cont)	PT vs.Cont	180.5	[123.0, 238.0]	<0.0001
	PT+IH vs. PT	-106.8	[-164.3, -49.3]	0.0018
CD86 intensity (A.U.)	PT vs.Cont	9.365	[6.92, 11.81]	<0.0001
	PT+IH vs. PT	-6.144	[-8.59, -3.7]	<0.0001
CD206 intensity	PT vs.Cont	0.015	[-0.775, 0.805]	0.9689

(A.U.)	PT+IH vs. PT	4.788	[3.998, 5.578]	<0.0001
CD206/β-actin (% of Cont)	PT vs.Cont	18.04	[-27.85, 63.93]	0.539
	PT+IH vs. PT	107.4	[61.47, 153.20]	0.0003
GBP2/β-actin (% of Cont)	PT vs.Cont	394.5	[257.10, 531.90]	<0.0001
	PT+IH vs. PT	-344.3	[-481.70, -206.90]	0.0002
CLCF1/β-actin (% of Cont)	PT vs.Cont	91.54	[29.43, 153.6]	0.0066
	PT+IH vs. PT	134.5	[72.44, 196.6]	0.0005
S100A10 intensity (A.U.)	PT vs.Cont	1.212	[-0.64, 3.06]	0.1870
	PT+IH vs. PT	6.716	[4.86, 8.57]	<0.0001
C3d intensity (A.U.)	PT vs.Cont	18.68	[15.55, 21.81]	<0.0001
	PT+IH vs. PT	-11.48	[-14.61, -8.35]	<0.0001
BrdU+NeuN+ Cells	PT vs.Cont	3.000	[1.66, 4.34]	0.0011
	PT+IH vs. PT	6.000	[3.68, 8.32]	0.0010
$Ki67^+$ cells	PT vs.Cont	2.0	[0.72, 3.28]	0.0039
	PT+IH vs. PT	5.5	[4.22, 6.78]	<0.0001
DCX^+NeuN^+ Cells	PT vs.Cont	1.750	[0.12, 3.38]	0.0364
	PT+IH vs. PT	7.250	[5.62, 8.88]	<0.0001
RECA1/Ki67 double labeled cells (ROI)	PT vs.Cont	2.125	[0.80, 3.45]	0.0032
	PT+IH vs. PT	3.625	[2.30, 4.95]	<0.0001
ATP levels	PT vs.Cont	-41.76	[-57.88, -25.64]	<0.0001
	PT+IH vs. PT	31.68	[15.56, 47.80]	0.0010
Relative Evans blue content (A.U.)	PT vs.Cont	0.3840	[0.294, 0.474]	<0.0001
	PT+IH vs. PT	-0.2069	[-0.297, -0.117]	0.0002
MFN1/β-actin（% of Cont）	PT vs.Cont	29.07	[16.08, 41.34]	0.0002
	PT+IH vs. PT	16.66	[4.39, 28.93]	0.0203
MFN2/β-actin (% of Cont)	PT vs.Cont	-28.08	[-40.85, -15.31]	0.0005
	PT+IH vs. PT	27.22	[14.45, 39.99]	0.0005
OPA1/α-tubulin (% of Cont)	PT vs.Cont	-38.23	[-61.60, -14.86]	0.0147
	PT+IH vs. PT	34.74	[11.37, 58.11]	0.0167
CD86 mRNA expression	PT vs.Cont	1.143	[0.601, 1.685]	0.0063
	PT+IH vs. PT	-1.062	[-1.604, -0.520]	0.0063
CD206 mRNA expression	PT vs.Cont	1.791	[-0.183, 3.765]	0.0682
	PT+IH vs. PT	2.593	[0.619, 4.567]	0.0362
Arg1 mRNA expression	PT vs.Cont	0.0421	[-1.230, 1.314]	0.9380
	PT+IH vs. PT	2.041	[0.770, 3.312]	0.0209
Body weight (g)	PT vs.Cont	-0.0875	[-2.07, 1.89]	0.9270
	PT+IH vs. PT	-1.108	[-2.99, 0.77]	0.4895

Supplementary Table 4. A priori power analysis for aged mouse stroke study

Item	Value/Parameter	Notes/Calculation Basis
Software version	G*Power 3.1.9.7	Gold-standard for statistical power analysis in biomedical research
Statistical test type	One-way fixed-effects omnibus ANOVA (between-subjects)	For 3-group comparison (Control/PT/PT+IH)
Effect size (f)	0.80 (large)	Conservative choice accounting for high biological variability in aged mouse cohorts
Alpha level (α)	0.05 (two-tailed)	International standard significance threshold for biomedical research
Statistical power (1−β)	0.80	International standard, ensuring 80% probability of detecting a true treatment effect
Number of groups (k)	3	Control, photothrombotic (PT) stroke, PT+intermittent hypoxia (IH)
Minimum sample size per group (biological replicates, n)	8 mice	G*Power standard output for the above parameter combination
Total minimum sample size (N)	24 mice	$k \times n = 3 \times 8$
Noncentrality parameter (λ)	15.3600	$\lambda = N \times f^2 = 24 \times 0.80^2 = 15.36$
Critical F value	3.4668	Derived from F-distribution table (df1=2, df2=21, α=0.05)
Numerator degrees of freedom (df1)	2	df1 = k-1 = 3-1
Denominator degrees of freedom (df2)	21	df2 = N-k = 24-3
Actual sample size (behavioral assays)	8-12 mice/group	Exceeds minimum to account for potential post-stroke attrition in aged mice
Actual sample size (histological/molecular analyses)	8 mice/group	Matches the minimum required sample size (statistically sufficient)
Replication type definition	n = biological replicates (mice)	Technical replicates (brain sections/ROIs) averaged per mouse to avoid pseudoreplication

Supplementary Table 5. GSEA of KEGG pathways in peri-infarct tissues

Comparison	Pathway	NES	P-value	FDR q-value
PT vs Cont	Apoptosis	1.5780	<0.0001	0.0118
PT vs Cont	Ferroptosis	1.6669	<0.0001	0.0040
PT vs Cont	Necroptosis	1.6508	<0.0001	0.0050
PT vs Cont	NF-κB signaling pathway	1.5527	<0.0001	0
PT vs Cont	Toll-like receptor signaling pathway	1.8462	<0.0001	3.11E-04
PT vs Cont	RIG-I-like receptor signaling pathway	1.6710	<0.0001	0.0040
PT vs Cont	TGF-β signaling pathway	1.6741	<0.0001	0.0040
PT vs Cont	JAK-STAT signaling pathway	1.5855	<0.0001	0
PT vs Cont	Natural killer cell-mediated cytotoxicity	1.5519	<0.0001	0
IH vs PT	Apoptosis	-1.6614	<0.0001	0.0230
IH vs PT	Ferroptosis	-1.6465	0.0090	0.0246
IH vs PT	Necroptosis	-1.6369	0.0012	0.0248
IH vs PT	NF-κB signaling pathway	-1.6313	0.0012	0.0250
IH vs PT	Toll-like receptor signaling pathway	-1.5671	0.0072	0.0426
IH vs PT	RIG-I-like receptor signaling pathway	-1.3740	0.0733	0.1234
IH vs PT	TGF-β signaling pathway	-1.6433	<0.0001	0.0241
IH vs PT	JAK-STAT signaling pathway	-1.7832	<0.0001	0.0109
IH vs PT	Natural killer cell-mediated cytotoxicity	-1.2305	0.1496	0.2670
